# Substituent Effects in Tetrel Bonds Involving Aromatic Silane Derivatives: An ab initio Study

**DOI:** 10.3390/molecules28052385

**Published:** 2023-03-05

**Authors:** Sergi Burguera, Antonio Frontera, Antonio Bauzá

**Affiliations:** Department of Chemistry, Universitat de les Illes Balears, Ctra. de Valldemossa km 7.5, 07122 Palma de Mallorca, Baleares, Spain

**Keywords:** silicon tetrel bonding interactions, ab initio calculations, substituent effects, aromatic silanes

## Abstract

In this manuscript substituent effects in several silicon tetrel bonding (TtB) complexes were investigated at the RI-MP2/def2-TZVP level of theory. Particularly, we have analysed how the interaction energy is influenced by the electronic nature of the substituent in both donor and acceptor moieties. To achieve that, several tetrafluorophenyl silane derivatives have been substituted at the meta and para positions by several electron donating and electron withdrawing groups (EDG and EWG, respectively), such as –NH_2_, –OCH_3_, –CH_3_, –H, –CF_3_ and –CN substituents. As electron donor molecules, we have used a series of hydrogen cyanide derivatives using the same EDGs and EWGs. We have obtained the Hammett’s plots for different combinations of donors and acceptors and in all cases we have obtained good regression plots (interaction energies vs. Hammet’s σ parameter). In addition, we have used the electrostatic potential (ESP) surface analysis as well as the Bader’s theory of atoms in molecules (AIM) and noncovalent interaction plot (NCI plot) techniques to further characterize the TtBs studied herein. Finally, a Cambridge Structural Database (CSD) inspection was carried out, retrieving several structures where halogenated aromatic silanes participate in tetrel bonding interactions, being an additional stabilization force of their supramolecular architectures.

## 1. Introduction

Noncovalent interactions (NCIs) are of crucial importance in molecular recognition and set the foundation of modern chemistry [1,2,3,4]. From the beginning of the century, NCIs have earned a general recognition that has moved the field far beyond classical and ubiquitous forces such as hydrogen bonding and π–π stacking interactions. Concretely, the σ-/π-hole crown of interactions [5], composed by halogen [6], chalcogen [7], pnictogen [8], tetrel [9] and aerogen bonds [10], has become a reliable and robust resource for driving molecular recognition [11,12] and self-assembly processes [13,14,15] with the assistance of computational chemistry [16,17], which is crucial for achieving a full exploitation of the NCIs in several fields (e.g., materials science, catalysis, biochemistry, etc.) [18,19,20,21,22,23].

The number of Investigations in NCIs [24,25] encompassing elements from groups 14–17 have exponentially grown during the past decade, leading to the creation and exploitation of novel fields of research [26,27,28]. Several computational studies [5,6,7,8,9,10,24,25] have demonstrated the anisotropic nature of the electron density distribution in Halogen (Ha), Chalcogen (Ch) and Pnictogen (Pn) elements owing to the coexistence of lone pairs (LPs) and σ–holes on the same atom. More precisely, in monovalent Ha, divalent Ch, trivalent Pn and tetravalent Tt atoms, the LP number goes from three to none, while the number of σ-holes goes from one to four (moving from group 17 to group 14). This is important, since the number and position of the positive and negative molecular electrostatic surface potentials on these atoms is closely related to the number and location of the covalent bonds the atoms are involved in, as well as the location of the LPs.

Among this number of NCIs, tetrel bonds (TtBs), which involve a σ-hole located on an element from group IV and a Lewis base, were theoretically described by the groups of Frontera [29] and Arunan [30] and have nowadays achieved great recognition in the fields of supramolecular chemistry and crystal engineering, as well as in biological systems [31,32,33,34,35,36,37,38,39]. In spite of this, Johnson and co-workers first showed the stability of carbon-Lewis base adducts [40]. They demonstrated that the geometrical disposition of the [H_2_O–CO_2_] dimer was ruled by a C⋯O interaction that was stronger than the dimer based on a H⋯O hydrogen bond (HB). This finding was confirmed by the group of Klemperer et al. several years later via microwave spectral analysis [41]. In more detail, they demonstrated the prevalence of a supramolecular complex featuring a O_2_C⋯OH_2_ tetrel bond instead of a hydrogen bond (HO–H⋯O=C=O). Simple calculations at B97D/6-31 + G* yielded a more stable tetrel bonded complex by 1.8 kcal/mol compared to the HB geometry. In addition, it was also demonstrated during the 1980s that other HB donor molecules, such as HBr [42] and HCN [43], formed stronger tetrel bonding complexes with carbon dioxide.

Later in 2001, Alkorta and collaborators calculated a series of supramolecular complexes between Si derivatives (SiX_4_, X = halogen) and lone pair donors (Z = NH_3_, H_2_O, etc.). The Si⋯Z interaction distances obtained from the calculations range between 2.1 and 4.1 Å, while the strength of the interaction reached up to −10 kcal/mol for the stronger complexes [44]. In a parallel work, Alkorta evaluated the conformational trend of aminopropylsilanes [45] by using NMR data from experiments (^15^N and ^29^Si chemical shifts and J_N–Si_ coupling constants) in combination with theoretical calculations. The results obtained provided evidence of the existence of an equilibrium between an open chain structure (dominated by the entropic component) and the supramolecular cycle (governed by the enthalpic term). Finally, the energetic and geometric features of TtBs in neutral and protonated RTtF_3_ (R = pyridinyl and furanyl) systems have been investigated by Scheiner and collaborators [46] for C, Si and Ge, using NH_3_ as a Lewis base.

The physical nature of the interaction is based on two main factors [47,48]. First, the polarizability of the tetrel atom (Tt), which increases upon descending in the group. Consequently, the electropositive region of the σ–hole increases if the EWG–Tt bond (EWG: electron withdrawing group) is more polarized, resulting in a strengthening of the NCI. Secondly, another way to polarize the EWG–Tt bond is by increasing the electron-withdrawing ability of the EWG. Therefore, the combination of heavy elements and a strong EWG increases the positive potential and size of the σ–hole, thus reinforcing the NCI (by increasing the contribution of electrostatics). In terms of steric demands, TtBs are a world apart compared to other σ-hole interactions. For instance, while the approaching LP donor molecule in a typical halogen bond (HalB) complex is usually separated by 180° from the EWG covalently attached to the halogen atom (with the minimal amount of steric repulsion), the tetrahedral spacing of the four substituents bound to the Tt atom dramatically increases the steric crowding and, at the same time, augments the steric repulsion with the Lewis base.

In this context, silicon is probably more prone to participate in noncovalent TtBs than the heavier tetrels due to their tendency to expand their valence, thus engaging in covalent/coordination chemistry. Moreover, tetrahedral lead(IV) compounds are quite uncommon, and lead(II) has a rich coordination chemistry [49,50,51,52] and it is considered a metal. Nonetheless, hypervalent species of Si are also known [53,54,55,56] although less abundant. The aim of this study is to investigate the geometric and energetic features of tetrel bonded complexes involving tetrafluorophenyl silane moieties and to study substituent effects in the meta and para positions of the ring. A similar study encompasses the work from Franconetti and collaborators [57], where substituent effects on a series of “like–like” Sn···Sn Tetrel bond complexes were evaluated at the RI-MP2/def2-TZVP level of theory. The authors used para-substituted phenylstannane derivatives as TtB donors and several tin-containing benzene derivatives as TtB acceptor species. In addition, the study from An and co-workers [58] shed light on the basis set influence (using double and triple zeta Dunning’s basis sets), substitution and competition in Tetrel bonding interactions involving phenyltrifluorosilane and dimethyl sulfoxide molecules. Building upon these previous works, we were interested in (i) evaluating meta-substitution effects on the aromatic silane moiety, (ii) including substitution on the electron donor molecule and (iii) inspecting the Cambridge Structural Database (CSD) [59] for specific examples of Si TtBs involving aromatic silanes.

**Figure 1:**
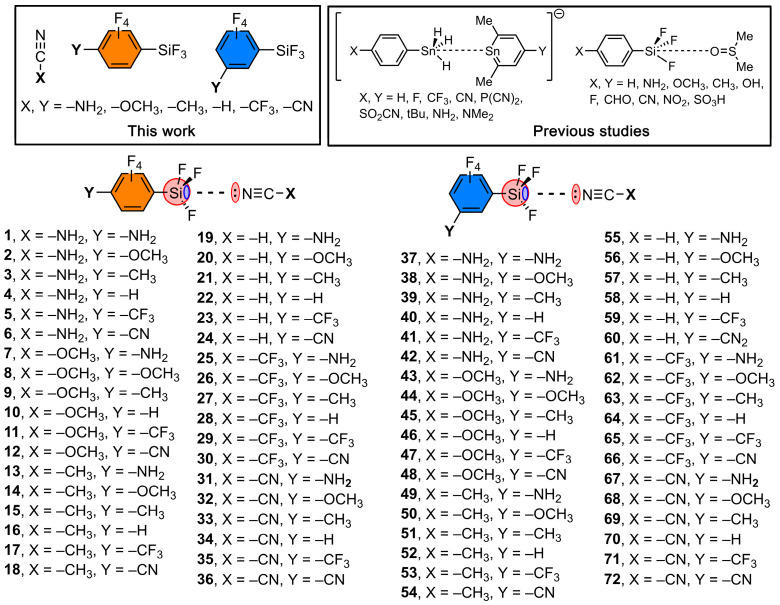
Compounds and complexes **1** to **72** studied in this work.

To achieve that, we have used several electron-donating and electron-withdrawing groups (EDG and EWG, respectively), such as –NH_2_, –OCH_3_, –CH_3_, –H, –CF_3_ and –CN substituents, incorporated at the meta and para positions of a fluorinated aromatic silane derivative (see Figure 1). As electron donor molecules, we have used a series of hydrogen cyanide derivatives using the same EDGs and EWGs (see ESI for cartesian coordinates of complexes **1** to **72** in supplementary material). In addition, the topological analysis of the electron density was carried out using the quantum theory of atoms in molecules (QTAIM) and noncovalent interactions plot (NCI plot) methodologies. Finally, a CSD inspection was performed, revealing some examples where halogenated aromatic silanes undergo TtBs in their solid-state structure, thus giving reliability to the results obtained from the calculations. We expect that the evidence reported herein will be useful for those scientists devoted to the fields of supramolecular chemistry and crystal engineering.

## 2. Results and Discussion

### 2.1. ESP Analysis

**Figure 2:**
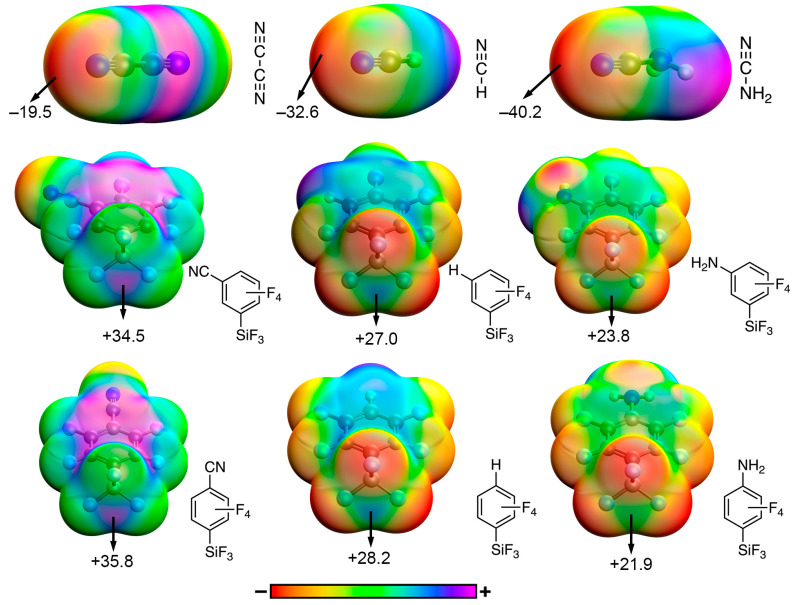
Electrostatic potential surfaces (ESP) of some selected compounds used in this study. The energy values given at selected points in the surface are in kcal/mol using an isovalue of 0.001 a.u. The ESP values corresponding to the rest of the compounds are gathered in Table 1.

We started our study by carrying out an electrostatic potential surface (ESP) analysis on the tetrel bond donor and acceptor molecules used herein (see Figure 2 for some representative examples). Compounds involving –CN, –H and –NH_2_ substituents are discussed in more detail while the complete list of ESP values is gathered in Table 1. Firstly, in the case of the three hydrogen cyanide derivatives, the basicity of the sp N atom increased continually from –CN to –NH_2_ substituents, following their electron-donating or electron-withdrawing nature. Secondly, in the case of the TtB donor moieties four σ-holes were found over the Si atom, due to the four established covalent bonds. Our interests focused on the σ-hole belonging to the C–Si bond, which is depicted in Figure 2, thus, the presence of a EWG or EDG group resulted in an increase or a decrease of the ESP value over the Si σ-hole, respectively. This was observed in both the meta- and para-substituted aromatic silanes, achieving the latter slightly more positive (meta-CN (+34.5 kcal/mol) and para-CN (+35.8 kcal/mol)) or less positive (meta-NH_2_ (+23.8 kcal/mol) and para-NH_2_ (+21.9 kcal/mol)) ESP values. These results are in line with previous studies on the substituent effects in halogen bonding interactions [36], pointing out that resonance effects are not an important factor affecting the Si σ-hole’s potential and that TtB complexes involving either meta- or para-substituted silanes are expected to show a similar strength. On the other hand, using –H as substituent (either on the TtB donor or acceptor molecule) middle point results were reported, as expected. Finally, the rest of the ESP values included in Table 1 follow the expected trends for both the TtB donor and acceptor molecules, depending on their electron-donating or electron-withdrawing nature.

### 2.2. Energetic Study

**Figure 3:**
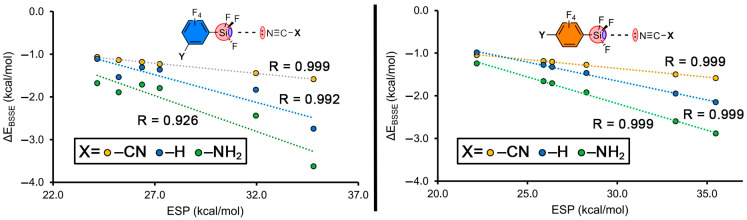
Electrostatic surface potential values (ESP) vs. the BSSE-corrected binding energies (ΔE_BSSE_) of several representative meta- (**left**) and para-substituted (**right**) complexes.

The values of the binding energies for all complexes of the study are included in Table 2 and Table 3, following the same behaviour independently of the position of the substituent in the aromatic ring. Firstly, in all the cases negative and attractive interaction energy values were obtained, ranging between −1.0 and −3.6 kcal/mol. If one focuses on the same electron donor molecule, those complexes involving EWGs (–CN and –CF_3_) resulted in more favourable binding energy values than those involving EDGs (–NH_2_, –OCH_3_ and –CH_3_), as expected from the ESP analysis discussed above. Besides, the position of the substituent on the ring (either meta or para) does not have a noticeable influence on the strength of the interaction, which is also in agreement with the ESP analysis shown above, indicating that the resonance effects do not play an important role in the stabilization of the Si tetrel bond complexes studied herein. Figure 3 shows a graphical representation of the ESP values vs. the binding energy values involving meta- and para-substituted aromatic silanes.

**Figure 4:**
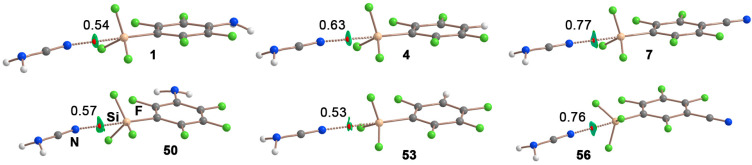
Distribution of critical points and bond paths in several tetrel bonding complexes. The intermolecular bond critical point (BCP) is denoted by a red dot. The bond path connecting the bond critical point is also represented. The value of the density at the BCP characterizing the TtB (ρ × 10^2^, a.u.) is also indicated. NCI plot colour range –0.002 a.u. ≤ (signλ_2_) ρ ≤ –0.002 a.u.

As noted, in both cases we found excellent correlations, thus reinforcing the ESP as a powerful predicting tool of the strength of these Si Tetrel bond complexes. Finally, in Figure 4 some optimized geometries at the RI-MP2/def2-TZVP level of theory are shown for six representative complexes exhibiting both meta and para substitution. In all the cases, a bond critical point (BCP) connects the sp N atom from the electron donor molecule with the Si atom of the aromatic silane moiety. In addition, a greenish isosurface can also be found along the bond path that connects both molecules, indicating the presence of the tetrel bonding interaction. The value of the density at the bond critical point that characterizes the TtBs is also given in Figure 4, while the rest of the values are also gathered in Table 2 and Table 3.

### 2.3. Hammett’s Representations

**Figure 5:**
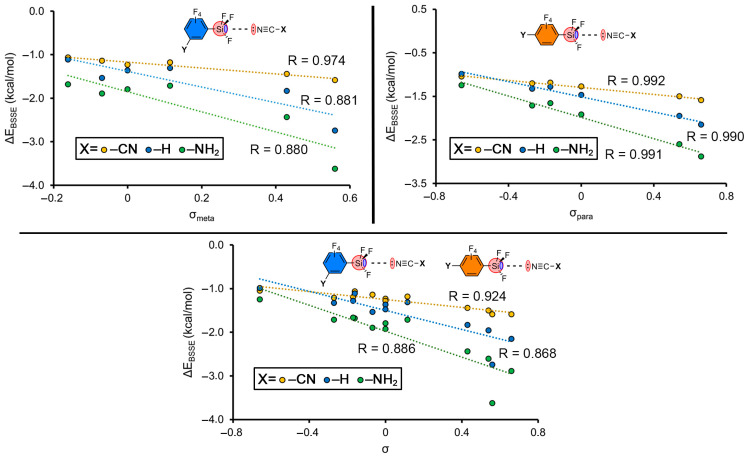
Hammett’s plots (ΔE_BSSE_ vs. σ) of meta- and para-substituted complexes involving –CN, –H, –NH_2_ groups (top left and right, respectively). Hammett’s plots (ΔE_BSSE_ vs. σ) including meta- and para-substituted complexes in the same representation (bottom).

**Figure 6:**
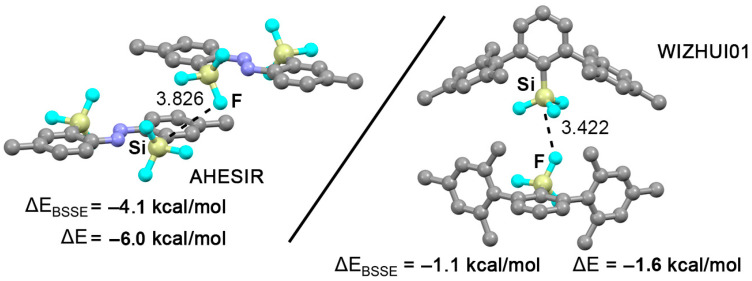
Partial views of the X-ray structures corresponding to AHESIR (left) and WIZHUI01 (middle). The tetrel bond distances are given in Å. The CSD codes are also indicated.

Figure 5 and Figure 6 gather several Hammett regression plots, where the σ_m_ and σ_p_ constants were plotted against the binding energies of those meta and para complexes involving –CN, –H and –NH_2_ as electron donor substituents. As noted, very good correlations were found in those representations that separately involve the meta- and para-substituted complexes (Figure 5, top). In addition, if these two plots were combined (Figure 5, bottom) the correlation was still very good, with R values of 0.918 (–CN), 0.856 (–H) and 0.874 (–H), thus showing that resonance effects do not play a significant role in the substituent effects involving these systems, similarly to previously published results [60].

### 2.4. CSD Search

As the last stage of our study, the CSD was inspected (December 2022) and five structures were found exhibiting tetrel bonding interactions between halogenated aromatic silane moieties. Three of them are shown in Figure 6, which correspond to AHESIR [61] and WIZHUI01 [62] X-ray structures. Firstly, the AHESIR structure corresponds to (E) 4,4′-Dimethyl-2,2′-disilylazobenzene, which exhibits discrete monomeric units interacting through several NCIs (e.g., π-π stacking, CH-π) on its solid state architecture. In addition to these, Si TtBs are undertaken between a F atom from the –SiF_3_ moiety and the Si–F σ-hole from a neighbouring unit. The WIZHUI01 structure involves a polymorph of the compound 2,6-Mes_2_C_6_H_3_SiF_3_, which shows discrete units self-assembling through F···Si TtBs bonds in the solid state architecture, forming a one-dimensional columnar pattern. In Figure 6 the interaction strength as well as the TtB distances are indicated, which lie within the same range as the results obtained using fully optimized models, thus giving reliability to the binding energies obtained.

## 3. Conclusions

The above computations indicate the ability of tetrafluorophenyl silanes to engage in TtB interactions. The substituent effects were evaluated by using a series of EDGs and EWGs on the meta and para positions of the ring and on the electron donor molecule and by correlating the Hammett substituent constants with the strength of the interaction, obtaining excellent correlations in all cases. These indicate that resonance effects do not play a crucial role and that substituent effects can be explained on the basis of inductive effects. In addition, a survey of the CSD revealed several X-ray structures where TtBs between halogenated aromatic silane moieties were undertaken, contributing to their stabilization of the supramolecular architectures. We expect that the results reported herein will be useful for those scientists devoted to the fields of supramolecular chemistry and crystal engineering of silicon-based compounds.

## 4. Materials and Methods

### 4.1. General Considerations

The interaction energies of all the complexes used were calculated at the RI-MP2 [63]/def2-TZVP [64] level of theory. This level of theory has achieved success to accurately represent interaction energies involving both neutral and charged electron donors [65]. The calculations were performed using the TURBOMOLE program, version 7.0. [66]. The interaction energies (ΔE_BSSE_, in kcal/mol) were calculated as the energy difference between the complex and the isolated monomers following the supermolecule approximation (ΔE_complex_ = E_complex_ − E_monomerA_ − E_monomerB_). These values were corrected using the Boys and Bernardi counterpoise correction method [67]. Frequency calculations demonstrated the true minima nature of the TtB complexes studied herein. We have also calculated the Gibbs free energies for two representative complexes (**18** and **54**) involving –CH_3_ and –CN groups and resulting in positive values, thus pointing to the importance of entropic effects when dealing with this family of compounds. In the case of the X-ray crystal models of AHESIR and WIZHUI01 structures, single point calculations were carried out to obtain the interaction energy values at the RI-MP2/def2-TZVP level of theory. The MEP surfaces were computed using Gaussian 16 software [68]. The Bader’s AIM theory [69] was used to analyse and describe the interactions discussed in this work using the AIMall calculation package [70]. The RI-MP2/def2-TZVP level of theory was also used for the wavefunction analysis (also using Gaussian 16 software). The NCI plot [71] isosurfaces acknowledge the presence of both attractive and repulsive interactions, as denoted by the sign of the second density Hessian eigenvalue and characterized by the isosurface colour. The colour scheme is composed of a red–yellow–green–blue scale using red for repulsive (λ^+^_cut_) and blue for attractive (λ^−^_cut_) NCI interaction density. Weak repulsive and weak attractive interactions are identified by yellow and green surfaces, respectively.

### 4.2. CSD Survey

**Figure 7:**
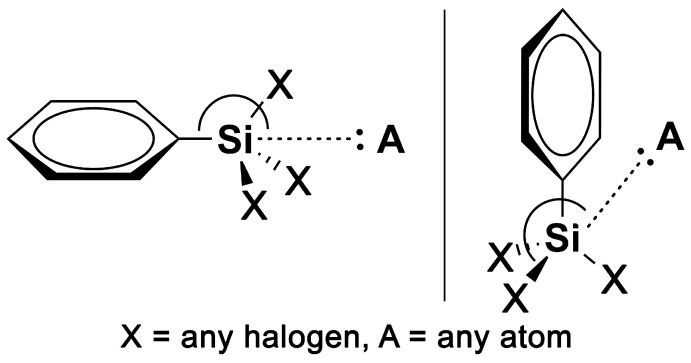
Conceptual models of the tetrel bonding interactions found in the CSD.

The CSD was inspected in December 22 (February 2021 update) using the Conquest program [72], retrieving five structures where tetrel bonds between halogenated aromatic silane moieties were undertaken. The following CSD codes from the structures were retrieved from the search: ASEHIR, EHIFAF, GOFSUR, PIDMOH and WIZHUI01. The geometrical criteria used to classify a noncovalent contact as TtB are as follows (see also Figure 7):

- d_Si···A_ ≤ sum of vdW radii + 0.5 Å.
- _C/X–Si···A_ between 160 and 180 degrees (X = C, F, Cl, Br and I and A = any atom).

## Figures and Tables

**Table 1:** Values of the electrostatic potential surface (ESP, in kcal/mol) for both tetrel bond donors (TtB donor, measured at the Si atom) and acceptor (TtB acceptor, measured at the N atom) molecules used in this study.

TtB Donor (para substitution)	ESP (kcal/mol)
**–CF_3_**	+33.3
**–CN**	+35.8
**–H**	+28.2
**–CH_3_**	+25.9
**–NH_2_**	+21.9
**–OCH_3_**	+26.4
**TtB donor (meta substitution)**	**ESP (kcal/mol)**
**–CF_3_**	+32.0
**–CN**	+34.5
**–H**	+27.0
**–CH_3_**	+25.2
**–NH_2_**	+23.8
**–OCH_3_**	+26.4
**TtB acceptor**	**ESP (kcal/mol)**
**–CF_3_**	−23.3
**–CN**	−19.5
**–H**	−32.6
**–CH_3_**	−38.3
**–NH_2_**	−40.2
**–OCH_3_**	−39.2

**Table 2:** Values of the uncorrected and BSSE-corrected interaction energies (ΔE and ΔE_BSSE_, in kcal/mol), equilibrium distances (d, in Å) and value of the density (ρ × 100, in a.u.) at the bond critical point (BCP) that characterizes the tetrel bonding interaction in complexes **1** to **36**.

Complex	ΔE	ΔE_BSSE_	d	ρ × 100
**1** (X = –NH_2_, Y = –NH_2_)	−1.9	−1.2	3.159	0.72
**2** (X = –NH_2_, Y = –OCH_3_)	−2.4	−1.7	3.114	0.77
**3** (X = –NH_2_, Y = –CH_3_)	−2.4	−1.7	3.119	0.77
**4** (X =–NH_2_, Y = –H)	−2.7	−1.9	3.095	0.79
**5** (X = –NH_2_, Y = –CF_3_)	−3.4	−2.6	3.030	0.89
**6** (X = –NH_2_, Y = –CN)	−3.7	−2.9	3.024	0.93
**7** (X = –OCH_3_, Y = –NH_2_)	−1.9	−1.2	3.173	0.69
**8** (X = –OCH_3_, Y = –OCH_3_)	−2.4	−1.7	3.142	0.72
**9** (X = –OCH_3_, Y = –CH_3_)	−2.3	−1.6	3.132	0.74
**10** (X = –OCH_3_, Y = –H)	−2.6	−1.9	3.108	0.77
**11** (X = –OCH_3_, Y = –CF_3_)	−3.3	−2.5	3.046	0.86
**12** (X = –OCH_3_, Y = –CN)	−3.6	−2.8	3.024	0.89
**13** (X = –CH_3_, Y = –NH_2_)	−1.9	−1.2	3.182	0.69
**14** (X = –CH_3_, Y = –OCH_3_)	−2.3	−1.6	3.139	0.74
**15** (X = –CH_3_, Y = –CH_3_)	−2.3	−1.6	3.143	0.74
**16** (X = –CH_3_, Y = –H)	−2.5	−1.8	3.121	0.77
**17** (X = –CH_3_, Y = –CF_3_)	−3.2	−2.4	3.060	0.85
**18** (X = –CH_3_, Y = –CN)	−3.5	−2.7	3.039	0.88
**19** (X = –H, Y = –NH_2_)	−1.5	−1.0	3.259	0.42
**20** (X = –H, Y = –OCH_3_)	−1.9	−1.3	3.221	0.64
**21** (X = –H, Y = –CH_3_)	−1.9	−1.3	3.218	0.64
**22** (X = –H, Y = –H)	−2.1	−1.5	3.206	0.66
**23** (X = –H, Y = –CF_3_)	−2.6	−2.0	3.155	0.71
**24** (X = –H, Y = –CN)	−2.8	−2.1	3.138	0.74
**25** (X = –CF_3_, Y = –NH_2_)	−1.5	−1.0	3.310	0.37
**26** (X = –CF_3_, Y = –OCH_3_)	−1.8	−1.2	3.268	0.58
**27** (X = –CF_3_, Y = –CH_3_)	−1.7	−1.1	3.283	0.57
**28** (X = –CF_3_, Y = –H)	−1.8	−1.3	3.267	0.58
**29** (X = –CF_3_, Y = –CF_3_)	−2.2	−1.6	3.228	0.62
**30** (X = –CF_3_, Y = –CN)	−2.3	−1.7	3.214	0.47
**31** (X = –CN, Y = –NH_2_)	−1.6	−1.0	3.324	0.69
**32** (X = –CN, Y = –OCH_3_)	−1.8	−1.2	3.297	0.74
**33** (X = –CN, Y = –CH_3_)	−1.8	−1.2	3.299	0.74
**34** (X = –CN, Y = –H)	−1.8	−1.3	3.286	0.77
**35** (X = –CN, Y = –CF_3_)	−2.1	−1.5	3.250	0.85
**36** (X = –CN, Y = –CN)	−2.2	−1.6	3.238	0.89

**Table 3:** Values of the uncorrected and BSSE-corrected interaction energies (ΔE and ΔE_BSSE_, in kcal/mol), equilibrium distances (d, in Å) and value of the density (ρ × 100, in a.u.) at the bond critical point (BCP) that characterizes the tetrel bonding interaction in complexes **37** to **72**.

Complex	ΔE	ΔE_BSSE_	d	ρ × 100
**37** (X = –NH_2_, Y = –NH_2_)	−2.4	−1.7	3.135	0.74
**38** (X = –NH_2_, Y = –OCH_3_)	−2.4	−1.7	3.112	0.77
**39** (X = –NH_2_, Y = –CH_3_)	−2.6	−1.9	3.131	0.75
**40** (X = –NH_2_, Y = –H)	−2.5	−1.8	3.162	0.71
**41** (X = –NH_2_, Y = –CF_3_)	−3.2	−2.4	3.048	0.87
**42** (X = –NH_2_, Y = –CN)	−3.6	−3.6	3.014	0.92
**43** (X = –OCH_3_, Y = –NH_2_)	−2.3	−1.6	3.160	0.71
**44** (X = –OCH_3_, Y = –OCH_3_)	−2.7	−2.0	3.128	0.75
**45** (X = –OCH_3_, Y = –CH_3_)	−2.6	−1.8	3.141	0.73
**46** (X = –OCH_3_, Y = –H)	−2.4	−1.7	3.142	0.73
**47** (X = –OCH_3_, Y = –CF_3_)	−3.1	−2.4	3.073	0.82
**48** (X = –OCH_3_, Y = –CN)	−3.5	−2.7	3.056	0.84
**49** (X = –CH_3_, Y = –NH_2_)	−2.0	−1.3	3.176	0.70
**50** (X = –CH_3_, Y = –OCH_3_)	−2.3	−1.6	3.164	0.71
**51** (X = –CH_3_, Y = –CH_3_)	−2.1	−1.5	3.157	0.72
**52** (X = –CH_3_, Y = –H)	−2.4	−1.7	3.121	0.77
**53** (X = –CH_3_, Y = –CF_3_)	−3.0	−2.3	3.070	0.84
**54** (X = –CH_3_, Y = –CN)	−3.4	−2.6	3.045	0.88
**55** (X = –H, Y = –NH_2_)	−1.7	−1.1	3.258	0.60
**56** (X = –H, Y = –OCH_3_)	−1.9	−1.3	3.232	0.63
**57** (X = –H, Y = –CH_3_)	−2.1	−1.5	3.238	0.62
**58** (X = –H, Y = –H)	−1.9	−1.4	3.223	0.64
**59** (X = –H, Y = –CF_3_)	−2.4	−1.8	3.168	0.70
**60** (X = –H, Y = –CN)	−2.7	−2.7	3.138	0.74
**61** (X = –CF_3_, Y = –NH_2_)	−1.9	−1.3	3.307	0.54
**62** (X = –CF_3_, Y = –OCH_3_)	−1.7	−1.2	3.287	0.56
**63** (X = –CF_3_, Y = –CH_3_)	−2.0	−1.4	3.290	0.56
**64** (X = –CF_3_, Y = –H)	−1.8	−1.2	3.280	0.57
**65** (X = –CF_3_, Y = –CF_3_)	−2.1	−1.5	3.229	0.62
**66** (X = –CF_3_, Y = –CN)	−2.3	−1.7	3.214	0.64
**67** (X = –CN, Y = –NH_2_)	−1.6	−1.1	3.341	0.51
**68** (X = –CN, Y = –OCH_3_)	−1.7	−1.2	3.302	0.55
**69** (X = –CN, Y = –CH_3_)	−1.7	−1.1	3.318	0.53
**70** (X = –CN, Y = –H)	−1.8	−1.2	3.308	0.54
**71** (X = –CN, Y = –CF_3_)	−2.0	−1.4	3.277	0.57
**72** (X = –CN, Y = –CN)	−2.2	−1.6	3.245	0.60

## Data Availability

All data to reproduce the results is gathered in the ESI in supplementary material.

## Supplementary Materials

The following supporting information can be downloaded at: https://www.mdpi.com/article/10.3390/molecules28052385/s1, Cartesian coordinates of complexes **1** to **72**.

## References

[1] Lehn J.M. (1995). Supramolecular Chemistry: Concepts and Perspectives.

[2] Schneider H.J. (2013). Supramolecular Systems in Biomedical Fields.

[3] Steed A.W., Atwood J.L. (2009). Supramolecular Chemistry.

[4] Cragg P.J. (2010). Supramolecular Chemistry: From Biological Inspiration to Biomedical Applications.

[5] Politzer P., Murray J. (2017). σ-Hole Interactions: Perspectives and Misconceptions. Crystals.

[6] Desiraju G.R., Ho P.S., Kloo L., Legon A.C., Marquardt R., Metrangolo P., Politzer P., Resnati G., Rissanen K. (2013). Definition of the halogen bond (IUPAC Recommendations 2013). Pure Appl. Chem..

[7] Aakeroy C.B., Bryce D.L., Desiraju G.R., Frontera A., Legon A.C., Nicotra F., Rissanen K., Scheiner S., Terraneo G., Metrangolo P. (2019). Definition of the chalcogen bond (IUPAC Recommendations 2019). Pure Appl. Chem..

[8] Zahn S., Frank R., Hey-Hawkins E., Kirchner B. (2011). Pnicogen bonds: A new molecular linker?. Chem. Eur. J..

[9] Bauzá A., Mooibroek T.J., Frontera A. (2013). Tetrel Bonding Interaction: Rediscovered Supramolecular Force?. Angew. Chem. Int. Ed..

[10] Bauzá A., Frontera A. (2020). σ/π-Hole noble gas bonding interactions: Insights from theory and experiment. Coord. Chem. Rev..

[11] Bauzá A., Frontera A., Mooibroek T.J. (2017). NO_3_^−^ anions can act as Lewis acid in the solid state. Nat. Commun..

[12] García-Llinás X., Bauzá A., Seth S.K., Frontera A. (2017). Importance of R–CF_3_···O Tetrel Bonding Interactions in Biological Systems. J. Phys. Chem. A.

[13] Bauzá A., Sharko A.V., Senchyk G.A., Rusanov E.B., Frontera A., Domasevitch K.V. (2017). π–hole interactions at work: Crystal engineering with nitro-derivatives. Cryst. Eng. Comm..

[14] Bauzá A., Frontera A., Mooibroek T.J. (2016). π-hole interactions involving nitro compounds: Directionality of nitrate esters. Cryst. Growth Des..

[15] Hazari A., Das L.K., Bauzá A., Frontera A., Ghosh A. (2014). The influence of H-bonding on the ‘ambidentate’coordination behaviour of the thiocyanate ion to Cd (II): A combined experimental and theoretical study. Dalton Trans..

[16] Politzer P., Murray J.S., Clark T. (2013). Halogen bonding and other σ-hole interactions: A perspective. Phys. Chem. Chem. Phys..

[17] Bauza A., Mooibroek T.J., Frontera A. (2015). The Bright Future of Unconventional σ/π-Hole Interactions. Chem. Phys. Chem..

[18] Riwar L.J., Trapp N., Root K., Zenobi R., Diederich F. (2018). Supramolecular Capsules: Strong versus Weak Chalcogen Bonding. Angew. Chem. Int. Ed..

[19] Wonner P., Dreger A., Vogel L., Engelage E., Huber S.M. (2019). Chalcogen Bonding Catalysis of a Nitro-Michael Reaction. Angew. Chem. Int. Ed..

[20] Borissov A., Marques I., Lim J.Y.C., Félix V., Smith M.D., Beer P.D. (2019). Anion Recognition in Water by Charge-Neutral Halogen and Chalcogen Bonding Foldamer Receptors. J. Am. Chem. Soc..

[21] Mallada B., Gallardo A., Lamanec M., de la Torre B., Špirko V., Hobza P., Jelinek P. (2021). Real-space imaging of anisotropic charge of σ-hole by means of Kelvin probe force microscopy. Science.

[22] Pascoe J., Ling K.B., Cockroft S.L. (2017). The Origin of Chalcogen-Bonding Interactions. J. Am. Chem. Soc..

[23] Macchione M., Goujon A., Strakova K., Humeniuk H.V., Licari G., Tajkhorshid E., Sakai N., Matile S. (2019). A Chalcogen-Bonding Cascade Switch for Planarizable Push-Pull Probes. Angew. Chem. Int. Ed..

[24] Politzer P., Murray J.S. (2019). An overview of strengths and directionalities of noncovalent interactions: σ-holes and π-holes. Crystals.

[25] Politzer P., Murray J.S., Clark T., Resnati G. (2017). The σ-hole revisited. Phys. Chem. Chem. Phys..

[26] Cavallo G., Metrangolo P., Pilati T., Resnati G., Terraneo G. (2014). Naming interactions from the electrophilic site. Cryst. Growth Des..

[27] Terraneo G., Resnati G. (2017). Bonding Matters. Cryst. Growth Des..

[28] Scheiner S. (2018). Steric crowding in tetrel bonds. J. Phys. Chem. A.

[29] Bauzá A., Mooibroek T.J., Frontera A. (2016). Tetrel Bonding Interactions. Chem. Rec..

[30] Mani D., Arunan E. (2013). The X–C⋯Y (X = O/F, Y = O/S/F/Cl/Br/N/P) ‘carbon bond’ and hydrophobic interactions. Phys. Chem. Chem. Phys..

[31] Sethio D., Oliveira V., Kraka E. (2018). Quantitative Assessment of Tetrel Bonding Utilizing Vibrational Spectroscopy. Molecules.

[32] Heywood V.L., Alford T.P.J., Roeleveld J.J., Deprez S.J.L., Verhoofstad A., van der Vlugt J.I., Domingos S.R., Schnell M., Davis A.P., Mooibroek T.J. (2020). Observations of tetrel bonding between sp^3^-carbon and THF. Chem. Sci..

[33] Zhang Y., Wang W., Wang Y.-B. (2019). Tetrel bonding on Graphene. Comp. Theor. Chem..

[34] Taylor M.S. (2020). Anion recognition based on halogen, chalcogen, pnictogen and tetrel bonding. Coord. Chem. Rev..

[35] Bauzá A., Seth S.K., Frontera A. (2019). Tetrel bonding interactions at work: Impact on tin and lead coordination compounds. Coord. Chem. Rev..

[36] Mundlapati V.R., Sahoo D.K., Bhaumik S., Jena S., Chandrakar A., Biswal H.S. (2018). Noncovalent Carbon-Bonding Interactions in Proteins. Angew. Chem. Int. Ed..

[37] Dutta J., Sahoo D.K., Jena S., Tulsiyan K.D., Biswal H.S. (2020). Non-covalent interactions with inverted carbon: A carbo-hydrogen bond or a new type of hydrogen bond?. Phys. Chem. Chem. Phys..

[38] Murray J.S., Lane P., Clark T., Riley K.E., Politzer P. (2012). σ-Holes, π-holes and electrostatically-driven interactions. J. Mol. Mod..

[39] Burgi H.B., Dunitz J.D., Shefter E. (1973). Geometrical reaction coordinates. II. Nucleophilic addition to a carbonyl group. J. Am. Chem. Soc..

[40] Jönsson B., Karlström G., Wennerström H. (1975). Ab initio molecular orbital calculations on the water-carbon dioxide system: Molecular complexes. Chem. Phys. Lett..

[41] Peterson K.I., Klemperer W. (1984). Structure and internal rotation of H_2_O−CO_2_, HDO−CO_2_ and D_2_O−CO_2_ van der Waals complexes. J. Chem. Phys..

[42] Peng Y.P., Sharpe S.W., Shin S.K., Wittig C., Beaudet R.A. (1992). Infrared spectroscopy of CO_2_–D(H)Br: Molecular structure and its reliability. J. Chem. Phys..

[43] Leopold K.R., Fraser G.T., Klemperer W. (1984). Rotational spectrum and structure of the complex HCNCO_2_. J. Chem. Phys..

[44] Alkorta I., Rozas I., Elguero J. (2001). Molecular Complexes between Silicon Derivatives and Electron-Rich Groups. J. Phys. Chem. A.

[45] Alkorta I., Elguero J., Fruchier A., Macquarrie D.J., Virgili A. (2001). Aminopropylsilanes versus silatranes: An experimental and theoretical study. J. Organomet. Chem..

[46] Liu M., Li Q., Scheiner S. (2017). Comparison of tetrel bonds in neutral and protonated complexes of pyridineTF_3_ and furanTF_3_ (T= C, Si, and Ge) with NH_3_. Phys. Chem. Chem. Phys..

[47] Murray J.S., Lane P., Politzer P. (2009). Expansion of the sigma-hole concept. J. Mol. Model..

[48] Murray J.S., Concha M.C., Politzer P. (2011). Molecular surface electrostatic potentials as guides to Si-O-N angle contraction: Tunable σ-holes. J. Mol. Model..

[49] Patia S., Rappoport Z. (1995). The Chemistry of Functional Groups. The Chemistry of Organic Germanium, Tin and Lead Compounds.

[50] Parr J., McCleverty J.A., Meye T. (2004). Comprehensive Coordination Chem II.

[51] Sato T., Abel E.W., Stone F.G.A., Wilkinson G. (1995). Comprehensive Organometallic Chem II—8—Tin.

[52] Pinhey J.T., Abel E.W., Stone F.G.A., Wilkinson G. (1995). 11—Lead. Comprehensive Organometallic Chem II.

[53] Greenberg A., Wu G. (1990). Structural relationships in silatrane molecules. Struct. Chem..

[54] Hencsei P. (1991). Evaluation of silatrane structures by correlation relationships. Struct. Chem..

[55] Voronkov M.G., Barishok V.P., Petukhov L.P., Rahklin R.G., Pestunovich V.A. (1988). 1-Halosilatranes. J. Organomet. Chem..

[56] Lukevics E., Dimens V., Pokrovska N., Zicmane I., Popelis J., Kemme A. (1999). Addition of nitrile oxides to 2,3-dihydrofurylsilanes. Crystal and molecular structure of tetrahydrofuro-[2,3-d]-isoxazolylsilanes. J. Organomet. Chem..

[57] Franconetti A., Frontera A. (2019). “Like–like” tetrel bonding interactions between Sn centres: A combined ab initio and CSD study. Dalton Trans..

[58] An X., Yang X., Li Q. (2021). Tetrel Bonds between Phenyltrifluorosilane and Dimethyl Sulfoxide: Influence of Basis Sets, Substitution and Competition. Molecules.

[59] Groom C.R., Bruno I.J., Lightfoot M.P., Ward S.C. (2016). The Cambridge Structural Database. Acta Cryst..

[60] Bauzá A., Quiñonero D., Frontera A., Deyà P.M. (2011). Substituent effects in halogen bonding complexes between aromatic donors and acceptors: A comprehensive ab initio study. Phys. Chem. Chem. Phys..

[61] Kano N., Yamamura M., Kawashima T. (2015). 2,2′-Disilylazobenzenes featuring double intramolecular nitrogen⋯silicon coordination: A photoisomerizable fluorophore. Dalton Trans..

[62] Schröder A., Lork E., Beckmann J. (2014). A monoclinic polymorph of 2,6-Mes_2_ C_6_ H_3_ SiF_3_. Main Group Met. Chem..

[63] Weigend F., Häser M. (1997). RI-MP2: First derivatives and global consistency. Theor. Chem. Acc..

[64] Weigend F., Ahlrichs R. (2005). Balanced basis sets of split valence, triple zeta valence and quadruple zeta valence quality for H to Rn: Design and assessment of accuracy. Phys. Chem. Chem. Phys..

[65] Bauzá A., Alkorta I., Frontera A., Elguero J. (2013). On the Reliability of Pure and Hybrid DFT Methods for the Evaluation of Halogen, Chalcogen, and Pnicogen Bonds Involving Anionic and Neutral Electron Donors. J. Chem. Theory Comput..

[66] Ahlrichs R., Bar M., Haser M., Horn H., Kolmel C. (1989). Electronic Structure Calculations on Workstation Computers—The Program System turbomole. Chem. Phys. Lett..

[67] Boys S.B., Bernardy F. (1970). The calculation of small molecular interactions by the differences of separate total energies. Some procedures with reduced errors. Mol. Phys..

[68] Frisch M.J., Trucks G.W., Schlegel H.B., Scuseria G.E., Robb M.A., Cheeseman J.R., Scalmani G., Barone V., Petersson G.A., Nakatsuji H. (2016). Gaussian 16, Revision B.01.

[69] Bader R.F.W. (1991). A quantum theory of molecular structure and its applications. Chem. Rev..

[70] Todd A., Keith T.K. (2013). AIMAll, version 13.05.06.

[71] Contreras-García J., Johnson E.R., Keinan S., Chaudret R., Piquemal J.-P., Beratan D.N., Yang W. (2011). NCIPLOT: A Program for Plotting Noncovalent Interaction Regions J. Chem. Theory Comput..

[72] Bruno I.J., Cole J.C., Edgington P.R., Kessler M., Macrae C.F., McCabe P., Pearson J., Taylor R. (2002). New software for searching the Cambridge Structural Database and visualising crystal structures. Acta Cryst..

