# Supplementary material for: Substituent Effects in Tetrel Bonds Involving Aromatic Silane Derivatives: An ab initio Study
Source: Molecules. 2023 Mar 5;28(5):2385. doi: 10.3390/molecules28052385 (PMC10004842; doi:10.3390/molecules28052385)
Supplement: Supplementary file 1 [file molecules-28-02385-s001.zip › molecules-2258691-supplementary.pdf]

Substituent Effects in Tetrel Bonds Involving Aromatic Silane Derivatives: An ab initio Study

Sergi Burguera, Antonio Frontera and Antonio Bauzá *

Department of Chemistry, Universitat de les Illes Balears, Ctra. de Valldemossa km 7.5, 07122 Palma de Mallorca, Baleares, Spain
* Correspondence: antonio.bauza@uib.es

Electronic Supplementary Information

Index

Cartesian coordinates of complexes 1 to 72 page 2
Cartesian coordinates of the selected CSD structures page 30

Cartesian coordinates of complexes 1 to 72

1.

N	0.18440970	-4.31102840	0.00000000
C	0.05755240	-5.47476450	0.00000000
N	-0.01602690	-6.81961140	0.00000000
H	-0.42961540	-7.20095420	0.84014280
H	-0.42961540	-7.20095420	-0.84014280
Si	0.13037350	-1.15213430	0.00000000
F	-0.60481300	-1.67840710	1.28700780
F	-0.60481300	-1.67840710	-1.28700780
F	1.63275850	-1.62173110	0.00000000
C	0.08180360	0.70350700	0.00000000
C	0.07289630	1.44485340	-1.18085610
C	0.07289630	1.44485340	1.18085610
C	0.04055320	2.82790990	-1.18704140
C	0.04055320	2.82790990	1.18704140
C	0.01682700	3.55900850	0.00000000
F	0.02069860	3.50272730	-2.34630550
F	0.02069860	3.50272730	2.34630550
F	0.10652680	0.82146900	-2.36591260
F	0.10652680	0.82146900	2.36591260
N	0.05422340	4.93585500	0.00000000
H	-0.27720710	5.37285130	-0.84719830
H	-0.27720710	5.37285130	0.84719830

2.

N	0.16086930	-4.77791980	0.00000000
C	0.04989660	-5.94315880	0.00000000
N	-0.00504090	-7.28822380	0.00000000
H	-0.41073210	-7.67687920	0.84058150
H	-0.41073210	-7.67687920	-0.84058150
Si	0.24014460	-1.66522060	0.00000000
F	-0.52266390	-2.14520280	1.28769710
F	-0.52266390	-2.14520280	-1.28769710
F	1.72175920	-2.19342970	0.00000000
C	0.28534330	0.19746510	0.00000000
C	0.31577220	0.93082930	-1.18474940
C	0.31577220	0.93082930	1.18474940
C	0.34784190	2.31795290	-1.19666790
C	0.34784190	2.31795290	1.19666790
C	0.36894020	3.03448340	0.00000000
F	0.36351920	2.97852340	-2.35303660
F	0.36351920	2.97852340	2.35303660
F	0.32175000	0.29875050	-2.36445560
F	0.32175000	0.29875050	2.36445560
O	0.46499900	4.38418380	0.00000000
C	-0.79719830	5.07739290	0.00000000
H	-0.84705150	5.69866760	0.89321940
H	-0.84705150	5.69866760	-0.89321940
H	-1.62658450	4.36914410	0.00000000

3.

N	0.15072110	-4.52718410	0.00000000
C	0.03704490	-5.69217750	0.00000000
N	-0.02109790	-7.03722430	0.00000000
H	-0.42846220	-7.42444900	0.84046130
H	-0.42846220	-7.42444900	-0.84046130
Si	0.09621600	-1.40912040	0.00000000
F	-0.64358220	-1.92448310	1.28753740
F	-0.64358220	-1.92448310	-1.28753740

F	1.60038110	-1.86992330	0.00000000
C	0.05727290	0.45338130	0.00000000
C	0.05566620	1.18974870	-1.18252430
C	0.05566620	1.18974870	1.18252430
C	0.02350280	2.57720810	-1.18311350
C	0.02350280	2.57720810	1.18311350
C	0.01070420	3.30774970	0.00000000
F	0.01548210	3.22396460	-2.35458620
F	0.01548210	3.22396460	2.35458620
F	0.09242360	0.56157420	-2.36393390
F	0.09242360	0.56157420	2.36393390
C	-0.02766530	4.80531030	0.00000000
H	-1.06020900	5.16182320	0.00000000
H	0.46328560	5.20011910	0.88767530
H	0.46328560	5.20011910	-0.88767530

4.

N	0.13653660	-3.70736390	0.00000000
C	0.04481850	-4.87422060	0.00000000
N	0.01228900	-6.21978150	0.00000000
H	-0.38638950	-6.61545840	0.84066980
H	-0.38638950	-6.61545840	-0.84066980
Si	0.07617480	-0.61300820	0.00000000
F	-0.66495480	-1.12330820	1.28811990
F	-0.66495480	-1.12330820	-1.28811990
F	1.58194470	-1.06686440	0.00000000
C	0.03904740	1.25288180	0.00000000
C	0.03781160	1.98351850	-1.18712850
C	0.03781160	1.98351850	1.18712850
C	0.00844820	3.37225360	-1.19076900
C	0.00844820	3.37225360	1.19076900
C	-0.00844430	4.07834590	0.00000000
F	0.00300510	4.02725880	-2.35418920
F	0.00300510	4.02725880	2.35418920
F	0.07511950	1.35037130	-2.36423260
F	0.07511950	1.35037130	2.36423260
H	-0.02844690	5.16074000	0.00000000

5.

N	0.07796360	-4.47587390	0.00000000
C	0.01793170	-5.64459480	0.00000000
N	0.02232240	-6.98953650	0.00000000
H	-0.36164050	-7.39810170	0.84129270
H	-0.36164050	-7.39810170	-0.84129270
Si	0.04960420	-1.44593670	0.00000000
F	-0.69986600	-1.93107050	1.29155940
F	-0.69986600	-1.93107050	-1.29155940
F	1.55346650	-1.90215570	0.00000000
C	0.03819000	0.42550930	0.00000000
C	0.04405060	1.16124270	-1.18101370
C	0.04405060	1.16124270	1.18101370
C	0.03248450	2.55054660	-1.19388200
C	0.03248450	2.55054660	1.19388200
C	0.04919660	3.27226640	0.00000000
F	-0.00110080	3.16681140	-2.37159480
F	-0.00110080	3.16681140	2.37159480
F	0.06069220	0.53576850	-2.35969440
F	0.06069220	0.53576850	2.35969440
C	0.02226510	4.78260140	0.00000000
F	0.63110990	5.28901650	-1.07307730
F	0.63110990	5.28901650	1.07307730

F	-1.24239980	5.22929330	0.00000000

6.

N	0.06761470	-3.98048640	0.00000000
C	0.02282070	-5.14982310	0.00000000
N	0.04481950	-6.49416150	0.00000000
H	-0.33209470	-6.90861510	0.84156980
H	-0.33209470	-6.90861510	-0.84156980
Si	0.03873540	-0.97397900	0.00000000
F	-0.71200250	-1.45314560	1.29265600
F	-0.71200250	-1.45314560	-1.29265600
F	1.54382220	-1.42534510	0.00000000
C	0.02765020	0.89985630	0.00000000
C	0.03711290	1.63030880	-1.18748070
C	0.03711290	1.63030880	1.18748070
C	0.02559200	3.01783330	-1.20113690
C	0.02559200	3.01783330	1.20113690
C	0.01782800	3.72869960	0.00000000
F	0.02904970	3.67297020	-2.35567470
F	0.02904970	3.67297020	2.35567470
F	0.06660950	0.99959710	-2.36184810
F	0.06660950	0.99959710	2.36184810
C	0.00821790	5.15239570	0.00000000
N	-0.00004230	6.32494610	0.00000000

7.

N	0.79703080	-3.68977150	0.00000000
C	0.63891580	-4.85253240	0.00000000
O	0.49729030	-6.13502210	0.00000000
C	-0.89418350	-6.55493760	0.00000000
H	-0.86349750	-7.63929200	0.00000000
H	-1.38635340	-6.18065660	0.89576590
H	-1.38635340	-6.18065660	-0.89576590
Si	0.48958870	-0.53146920	0.00000000
F	-0.20199870	-1.12018190	1.28460430
F	-0.20199870	-1.12018190	-1.28460430
F	2.02419400	-0.87952470	0.00000000
C	0.28834980	1.31297690	0.00000000
C	0.21883740	2.05089750	-1.18100430
C	0.21883740	2.05089750	1.18100430
C	0.07386480	3.42665380	-1.18717200
C	0.07386480	3.42665380	1.18717200
C	-0.00921320	4.15335770	0.00000000
F	-0.00107950	4.09761130	-2.34630480
F	-0.00107950	4.09761130	2.34630480
F	0.30287890	1.43178740	-2.36573980
F	0.30287890	1.43178740	2.36573980
N	-0.08406750	5.52835940	0.00000000
H	-0.44835350	5.93781650	-0.84741500
H	-0.44835350	5.93781650	0.84741500

8.

N	0.71330890	-4.17047660	0.00000000
C	0.59681560	-5.33805090	0.00000000
O	0.50346430	-6.62446250	0.00000000
C	-0.87044990	-7.09958980	0.00000000
H	-0.79604660	-8.18180650	0.00000000
H	-1.37710160	-6.74555470	0.89587690
H	-1.37710160	-6.74555470	-0.89587690
Si	0.59432330	-1.03069600	0.00000000
F	-0.13592040	-1.56497150	1.28550120

F	-0.13592040	-1.56497150	-1.28550120
F	2.10615440	-1.46358700	0.00000000
C	0.50994980	0.82983670	0.00000000
C	0.48601540	1.56319830	-1.18497990
C	0.48601540	1.56319830	1.18497990
C	0.41384520	2.94881330	-1.19684480
C	0.41384520	2.94881330	1.19684480
C	0.38039930	3.66471380	0.00000000
F	0.37955290	3.60887460	-2.35297970
F	0.37955290	3.60887460	2.35297970
F	0.53947980	0.93276870	-2.36428130
F	0.53947980	0.93276870	2.36428130
O	0.37296090	5.01769300	0.00000000
C	-0.93877540	5.61213160	0.00000000
H	-1.03618780	6.22780690	0.89313320
H	-1.03618780	6.22780690	-0.89313320
H	-1.71147160	4.84242290	0.00000000

9.

N	0.67991560	-3.90294920	0.00000000
C	0.58618110	-5.07252500	0.00000000
O	0.51651020	-6.36041410	0.00000000
C	-0.84913960	-6.85895900	0.00000000
H	-0.75645590	-7.93974250	0.00000000
H	-1.36157270	-6.51332520	0.89587020
H	-1.36157270	-6.51332520	-0.89587020
Si	0.41185330	-0.78253100	0.00000000
F	-0.29350790	-1.34975750	1.28536820
F	-0.29350790	-1.34975750	-1.28536820
F	1.94233750	-1.14505540	0.00000000
C	0.24852830	1.07243770	0.00000000
C	0.19798760	1.80681780	-1.18267460
C	0.19798760	1.80681780	1.18267460
C	0.07395900	3.18907920	-1.18322110
C	0.07395900	3.18907920	1.18322110
C	0.01291260	3.91707290	0.00000000
F	0.02292480	3.83384820	-2.35458090
F	0.02292480	3.83384820	2.35458090
F	0.27595670	1.18195010	-2.36376930
F	0.27595670	1.18195010	2.36376930
C	-0.12444240	5.40880900	0.00000000
H	-1.17831740	5.69619610	0.00000000
H	0.33931090	5.83521770	0.88767630
H	0.33931090	5.83521770	-0.88767630

10.

N	0.59557830	-3.06198460	0.00000000
C	0.56337880	-4.23477580	0.00000000
O	0.56212160	-5.52421920	0.00000000
C	-0.77495470	-6.09551710	0.00000000
H	-0.62410670	-7.16970210	0.00000000
H	-1.30507920	-5.77794660	0.89593390
H	-1.30507920	-5.77794660	-0.89593390
Si	0.33746380	0.03569580	0.00000000
F	-0.37333080	-0.52119210	1.28613370
F	-0.37333080	-0.52119210	-1.28613370
F	1.86649720	-0.33118550	0.00000000
C	0.18958740	1.89536890	0.00000000
C	0.14503740	2.62439610	-1.18727310
C	0.14503740	2.62439610	1.18727310
C	0.03364410	4.00895850	-1.19087190

C	0.03364410	4.00895850	1.19087190
C	-0.02487740	4.71270390	0.00000000
F	-0.01065990	4.66247100	-2.35418150
F	-0.01065990	4.66247100	2.35418150
F	0.21941250	1.99410270	-2.36408220
F	0.21941250	1.99410270	2.36408220
H	-0.10873660	5.79203620	0.00000000

11.

N	0.45963730	-3.84878920	0.00000000
C	0.45814480	-5.02179370	0.00000000
O	0.49218870	-6.30989760	0.00000000
C	-0.82828420	-6.92006100	0.00000000
H	-1.36697000	-6.61800460	-0.89611130
H	-0.64571380	-7.98924540	0.00000000
H	-1.36697000	-6.61800460	0.89611130
Si	0.29592720	-0.80694050	0.00000000
F	-0.43528970	-1.32467810	1.28944500
F	-0.43528970	-1.32467810	-1.28944500
F	1.81521110	-1.20834420	0.00000000
C	0.21217730	1.06189300	0.00000000
C	0.18963680	1.79701110	-1.18117960
C	0.18963680	1.79701110	1.18117960
C	0.12451860	3.18482880	-1.19398710
C	0.12451860	3.18482880	1.19398710
C	0.11343900	3.90653420	0.00000000
F	0.06706310	3.79933300	-2.37158820
F	0.06706310	3.79933300	2.37158820
F	0.23001220	1.17215210	-2.35954040
F	0.23001220	1.17215210	2.35954040
C	0.02820260	5.41479610	0.00000000
F	0.61698280	5.94425220	-1.07307670
F	0.61698280	5.94425220	1.07307670
F	-1.25283750	5.81205900	0.00000000

12.

N	0.39371700	-3.33227350	0.00000000
C	0.44948440	-4.50387900	0.00000000
O	0.54669380	-5.78837650	0.00000000
C	-0.74193550	-6.46372620	0.00000000
H	-1.29482920	-6.18889890	-0.89618780
H	-0.50606080	-7.52240200	0.00000000
H	-1.29482920	-6.18889890	0.89618780
Si	0.24498590	-0.31171430	0.00000000
F	-0.49133150	-0.81785350	1.29068400
F	-0.49133150	-0.81785350	-1.29068400
F	1.76204780	-0.72074840	0.00000000
C	0.17666940	1.56017690	0.00000000
C	0.16355670	2.29028080	-1.18763190
C	0.16355670	2.29028080	1.18763190
C	0.10974500	3.67679860	-1.20123550
C	0.10974500	3.67679860	1.20123550
C	0.08029210	4.38700800	0.00000000
F	0.09302130	4.33170920	-2.35567430
F	0.09302130	4.33170920	2.35567430
F	0.21189490	1.66032170	-2.36172060
F	0.21189490	1.66032170	2.36172060
C	0.02711370	5.80973690	0.00000000
N	-0.01712190	6.98148250	0.00000000

13.

N	-0.00931130	-3.99613920	0.00000000
C	0.00743060	-5.16372670	0.00000000
C	0.02880590	-6.61992510	0.00000000
H	-0.47748820	-6.99941870	0.88659410
H	1.05790900	-6.97678090	0.00000000
H	-0.47748820	-6.99941870	-0.88659410
Si	-0.00482880	-0.81380840	0.00000000
F	-0.75309810	-1.32162390	1.28654500
F	-0.75309810	-1.32162390	-1.28654500
F	1.48220080	-1.33293780	0.00000000
C	0.00405290	1.04154130	0.00000000
C	0.01713590	1.78262460	-1.18103560
C	0.01713590	1.78262460	1.18103560
C	0.02571970	3.16596770	-1.18716790
C	0.02571970	3.16596770	1.18716790
C	0.02347710	3.89739880	0.00000000
F	0.02600940	3.84108830	-2.34629350
F	0.02600940	3.84108830	2.34629350
F	0.03236440	1.15813460	-2.36580190
F	0.03236440	1.15813460	2.36580190
N	0.10125140	5.27227700	0.00000000
H	-0.21613670	5.71927790	-0.84733220
H	-0.21613670	5.71927790	0.84733220

14.

N	0.05583990	-4.47368170	0.00000000
C	0.03394920	-5.64105140	0.00000000
C	0.00728020	-7.09706670	0.00000000
H	1.02413080	-7.48745340	0.00000000
H	-0.51127280	-7.45945860	-0.88665630
H	-0.51127280	-7.45945860	0.88665630
Si	0.15476580	-1.33665450	0.00000000
F	-0.61283620	-1.81039420	1.28692130
F	-0.61283620	-1.81039420	-1.28692130
F	1.62778690	-1.89060000	0.00000000
C	0.22710960	0.52419540	0.00000000
C	0.26733520	1.25681070	-1.18495290
C	0.26733520	1.25681070	1.18495290
C	0.31804190	2.64334170	-1.19679060
C	0.31804190	2.64334170	1.19679060
C	0.34874090	3.35944860	0.00000000
F	0.34266030	3.30360930	-2.35301790
F	0.34266030	3.30360930	2.35301790
F	0.26476570	0.62425580	-2.36432370
F	0.26476570	0.62425580	2.36432370
O	0.46307830	4.70755550	0.00000000
C	-0.78966170	5.41804030	0.00000000
H	-0.83089320	6.03988080	0.89323420
H	-0.83089320	6.03988080	-0.89323420
H	-1.62862190	4.72117660	0.00000000

15.

N	0.00798020	-4.21861540	0.00000000
C	0.02295580	-5.38611000	0.00000000
C	0.04179490	-6.84226370	0.00000000
H	-0.46521200	-7.22066270	0.88664290
H	-0.46521200	-7.22066270	-0.88664290
H	1.07030220	-7.20080100	0.00000000
Si	-0.01834430	-1.07541450	0.00000000
F	-0.76571540	-1.58059400	1.28687580
F	-0.76571540	-1.58059400	-1.28687580

F	1.47588270	-1.57025810	0.00000000
C	-0.01930450	0.78653680	0.00000000
C	-0.00627730	1.52251480	-1.18272820
C	-0.00627730	1.52251480	1.18272820
C	-0.01082520	2.91031620	-1.18323420
C	-0.01082520	2.91031620	1.18323420
C	-0.00911450	3.64084270	0.00000000
F	-0.00589320	3.55707130	-2.35456310
F	-0.00589320	3.55707130	2.35456310
F	0.01796840	0.89329260	-2.36379900
F	0.01796840	0.89329260	2.36379900
C	-0.01799100	5.13885440	0.00000000
H	-1.04337670	5.51545120	0.00000000
H	0.48056240	5.52395060	0.88769680
H	0.48056240	5.52395060	-0.88769680

16.

N	0.00056540	-3.39218910	0.00000000
C	0.00999280	-4.55966390	0.00000000
C	0.02187690	-6.01583980	0.00000000
H	-0.48697410	-6.39168970	0.88666250
H	-0.48697410	-6.39168970	-0.88666250
H	1.04868170	-6.37919700	0.00000000
Si	-0.02314720	-0.27138640	0.00000000
F	-0.77253380	-0.77073670	1.28731150
F	-0.77253380	-0.77073670	-1.28731150
F	1.47204640	-0.76163930	0.00000000
C	-0.01974640	1.59387160	0.00000000
C	-0.00556170	2.32405380	-1.18734790
C	-0.00556170	2.32405380	1.18734790
C	-0.00549170	3.71308490	-1.19090510
C	-0.00549170	3.71308490	1.19090510
C	-0.00736420	4.41921030	0.00000000
F	0.00307280	4.36802820	-2.35415800
F	0.00307280	4.36802820	2.35415800
F	0.01827780	1.68977890	-2.36408500
F	0.01827780	1.68977890	2.36408500
H	-0.00448440	5.50179470	0.00000000

17.

N	-0.00209310	-4.17509790	0.00000000
C	0.00713510	-5.34235830	0.00000000
C	0.01872950	-6.79840880	0.00000000
H	0.53364610	-7.16574760	-0.88672230
H	0.53364610	-7.16574760	0.88672230
H	-1.00218090	-7.17799520	0.00000000
Si	-0.02253920	-1.11478140	0.00000000
F	-0.77448500	-1.59851050	1.29031600
F	-0.77448500	-1.59851050	-1.29031600
F	1.47578780	-1.59129940	0.00000000
C	-0.01474020	0.75545900	0.00000000
C	-0.00125940	1.49073570	-1.18125390
C	-0.00125940	1.49073570	1.18125390
C	0.00163110	2.88006020	-1.19400180
C	0.00163110	2.88006020	1.19400180
C	0.02598020	3.60141780	0.00000000
F	-0.02552980	3.49660130	-2.37156620
F	-0.02552980	3.49660130	2.37156620
F	0.00874910	0.86457490	-2.35956300
F	0.00874910	0.86457490	2.35956300
C	0.01494610	5.11210480	0.00000000

F	0.62917900	5.61184110	-1.07308040
F	0.62917900	5.61184110	1.07308040
F	-1.24488760	5.57184920	0.00000000

18.

N	-0.00842920	-3.67399510	0.00000000
C	0.00681060	-4.84111110	0.00000000
C	0.02580580	-6.29698740	0.00000000
H	-0.48126490	-6.67491690	0.88677260
H	-0.48126490	-6.67491690	-0.88677260
H	1.05450460	-6.65488480	0.00000000
Si	-0.02797820	-0.63544820	0.00000000
F	-0.78106140	-1.11367330	1.29139020
F	-0.78106140	-1.11367330	-1.29139020
F	1.47130060	-1.10767320	0.00000000
C	-0.02088130	1.23707250	0.00000000
C	-0.00506070	1.96708190	-1.18772160
C	-0.00506070	1.96708190	1.18772160
C	-0.00404420	3.35463420	-1.20127400
C	-0.00404420	3.35463420	1.20127400
C	-0.00542090	4.06540460	0.00000000
F	0.00534340	4.00966890	-2.35564180
F	0.00534340	4.00966890	2.35564180
F	0.01865340	1.33559970	-2.36170400
F	0.01865340	1.33559970	2.36170400
C	-0.00183200	5.48912810	0.00000000
N	0.00098880	6.66170590	0.00000000

19.

N	0.05602750	-5.12480770	0.00000000
C	0.08258300	-6.28984300	0.00000000
H	0.10720530	-7.35682090	0.00000000
Si	0.02486950	-1.86639760	0.00000000
F	-0.71524580	-2.39479200	1.28349640
F	-0.71524580	-2.39479200	-1.28349640
F	1.51325830	-2.38190150	0.00000000
C	0.01144100	-0.01391920	0.00000000
C	0.01495910	0.72667540	-1.18154090
C	0.01495910	0.72667540	1.18154090
C	0.00627300	2.10984800	-1.18753280
C	0.00627300	2.10984800	1.18753280
C	-0.00511030	2.84101860	0.00000000
F	-0.00192510	2.78490750	-2.34625420
F	-0.00192510	2.78490750	2.34625420
F	0.03752800	0.10119110	-2.36545550
F	0.03752800	0.10119110	2.36545550
N	0.05521500	4.21601150	0.00000000
H	-0.26433400	4.66049990	-0.84777030
H	-0.26433400	4.66049990	0.84777030

20.

N	0.05217260	-5.57655090	0.00000000
C	0.02455410	-6.74142310	0.00000000
H	-0.00035710	-7.80846240	0.00000000
Si	0.15667490	-2.35737910	0.00000000
F	-0.60886690	-2.84442760	1.28352230
F	-0.60886690	-2.84442760	-1.28352230
F	1.62441890	-2.92576560	0.00000000
C	0.22851260	-0.49950210	0.00000000
C	0.26831160	0.23236140	-1.18551220
C	0.26831160	0.23236140	1.18551220

C	0.31882060	1.61881130	-1.19712550
C	0.31882060	1.61881130	1.19712550
C	0.34949390	2.33469030	0.00000000
F	0.34326610	2.27900930	-2.35293580
F	0.34326610	2.27900930	2.35293580
F	0.26518060	-0.40153840	-2.36393410
F	0.26518060	-0.40153840	2.36393410
O	0.46419000	3.68232560	0.00000000
C	-0.78820100	4.39407230	0.00000000
H	-0.82858240	5.01583110	0.89326460
H	-0.82858240	5.01583110	-0.89326460
H	-1.62771760	3.69790080	0.00000000

21.

N	-0.00150630	-5.32781020	-0.00025600
C	0.00932850	-6.49297380	-0.00073450
H	0.01936180	-7.56024430	-0.00118800
Si	-0.01564220	-2.10944360	0.00027550
F	-0.76057860	-2.62509630	1.28474000
F	-0.76337800	-2.62489970	-1.28265420
F	1.47231380	-2.62358620	-0.00145360
C	-0.01119660	-0.25032100	0.00044800
C	0.00353050	0.48464200	-1.18292550
C	0.00305630	0.48502930	1.18360160
C	0.00088750	1.87238030	-1.18356680
C	0.00024530	1.87276650	1.18363450
C	0.00367750	2.60271480	-0.00008790
F	0.00735120	2.51873260	-2.35461800
F	0.00619880	2.51965950	2.35439020
F	0.02681170	-0.14621420	-2.36293970
F	0.02586680	-0.14524450	2.36390490
C	-0.00038370	4.10080880	-0.00016830
H	-1.02459620	4.48051310	0.00183200
H	0.50100350	4.48433050	0.88657860
H	0.49764830	4.48425650	-0.88881300

22.

N	0.00119270	-4.54448330	0.00000000
C	0.01398460	-5.70955040	0.00000000
H	0.02575910	-6.77684000	0.00000000
Si	-0.01899980	-1.33883790	0.00000000
F	-0.76605240	-1.85121040	1.28387490
F	-0.76605240	-1.85121040	-1.28387490
F	1.47082740	-1.84568090	0.00000000
C	-0.01480140	0.52345220	0.00000000
C	-0.00086860	1.25280200	-1.18792510
C	-0.00086860	1.25280200	1.18792510
C	-0.00075430	2.64179430	-1.19126350
C	-0.00075430	2.64179430	1.19126350
C	-0.00264400	3.34746710	0.00000000
F	0.00762630	3.29663440	-2.35406330
F	0.00762630	3.29663440	2.35406330
F	0.02232860	0.61717000	-2.36368070
F	0.02232860	0.61717000	2.36368070
H	0.00012210	4.43009250	0.00000000

23.

N	0.00321720	-5.29365340	0.00000000
C	0.01532500	-6.45852210	0.00000000
H	0.02642910	-7.52592930	0.00000000
Si	-0.01709640	-2.13883900	0.00000000

F	-0.76570800	-2.63876560	1.28629780
F	-0.76570800	-2.63876560	-1.28629780
F	1.47571770	-2.63266270	0.00000000
C	-0.01046930	-0.27195510	0.00000000
C	0.00180720	0.46242070	-1.18189110
C	0.00180720	0.46242070	1.18189110
C	0.00329220	1.85171880	-1.19436530
C	0.00329220	1.85171880	1.19436530
C	0.02699630	2.57268260	0.00000000
F	-0.02468750	2.46808700	-2.37151220
F	-0.02468750	2.46808700	2.37151220
F	0.01166480	-0.16520520	-2.35915780
F	0.01166480	-0.16520520	2.35915780
C	0.01488810	4.08379260	0.00000000
F	0.62879110	4.58339920	-1.07308780
F	0.62879110	4.58339920	1.07308780
F	-1.24532740	4.54177670	0.00000000

24.

N	-0.00174030	-4.82511410	0.00000000
C	0.01464500	-5.98985120	0.00000000
H	0.02958590	-7.05724880	0.00000000
Si	-0.02134790	-1.68754550	0.00000000
F	-0.77088860	-2.18311960	1.28703030
F	-0.77088860	-2.18311960	-1.28703030
F	1.47217400	-2.17795290	0.00000000
C	-0.01516990	0.18143900	0.00000000
C	-0.00031630	0.91046960	-1.18839780
C	-0.00031630	0.91046960	1.18839780
C	-0.00014170	2.29797360	-1.20167050
C	-0.00014170	2.29797360	1.20167050
C	-0.00191720	3.00832420	0.00000000
F	0.00854880	2.95291230	-2.35557060
F	0.00854880	2.95291230	2.35557060
F	0.02292580	0.27738380	-2.36126470
F	0.02292580	0.27738380	2.36126470
C	0.00069370	4.43205960	0.00000000
N	0.00282050	5.60465050	0.00000000

25.

N	-0.00836980	-4.06755130	0.00000000
C	0.00767680	-5.23552610	0.00000000
C	0.02813690	-6.71486680	0.00000000
F	1.28140140	-7.15764030	0.00000000
F	-0.58905410	-7.18341890	-1.08000630
F	-0.58905410	-7.18341890	1.08000630
Si	-0.00225460	-0.75706500	0.00000000
F	-0.74607800	-1.28936170	1.28046610
F	-0.74607800	-1.28936170	-1.28046610
F	1.47615450	-1.30305670	0.00000000
C	0.00527330	1.09222700	0.00000000
C	0.01685320	1.83207380	-1.18210910
C	0.01685320	1.83207380	1.18210910
C	0.02378280	3.21503450	-1.18797120
C	0.02378280	3.21503450	1.18797120
C	0.02064250	3.94616980	0.00000000
F	0.02329510	3.89011440	-2.34620590
F	0.02329510	3.89011440	2.34620590
F	0.03191710	1.20494760	-2.36507910
F	0.03191710	1.20494760	2.36507910
N	0.09595010	5.31927110	0.00000000

H	-0.21302170	5.76962940	-0.84846120
H	-0.21302170	5.76962940	0.84846120

26.

N	0.05378020	-4.55008690	-0.00022520
C	0.02674020	-5.71772210	-0.00001130
C	-0.00745950	-7.19723460	0.00025930
F	1.22853270	-7.68537650	-0.01270380
F	-0.65282400	-7.64194120	-1.07309770
F	-0.63022450	-7.64207230	1.08682930
Si	0.16062650	-1.28340350	-0.00020410
F	-0.60328480	-1.78326950	1.28009160
F	-0.60267830	-1.78331110	-1.28085360
F	1.62369490	-1.86533370	0.00016940
C	0.23176070	0.57142040	-0.00027460
C	0.27121350	1.30248350	-1.18638000
C	0.27099200	1.30249110	1.18583600
C	0.32130540	2.68884850	-1.19776640
C	0.32108760	2.68884890	1.19723190
C	0.35185730	3.40450710	-0.00026640
F	0.34569350	3.34888610	-2.35311810
F	0.34523230	3.34891270	2.35257930
F	0.26777540	0.66713190	-2.36382440
F	0.26739650	0.66711300	2.36327330
O	0.46706790	4.75154170	-0.00027430
C	-0.78486750	5.46489410	0.00053600
H	-0.82363540	6.08649270	0.89389120
H	-0.82471350	6.08659000	-0.89270520
H	-1.62506930	4.76958990	0.00100750

27.

N	-0.00307450	-4.30031680	0.00000000
C	0.00808780	-5.46823660	0.00000000
C	0.02235510	-6.94800030	0.00000000
F	1.27383190	-7.39542650	0.00000000
F	-0.59674120	-7.41347460	-1.08002020
F	-0.59674120	-7.41347460	1.08002020
Si	-0.01704040	-1.01718740	0.00000000
F	-0.76081730	-1.54634990	1.28038020
F	-0.76081730	-1.54634990	-1.28038020
F	1.46685030	-1.54469630	0.00000000
C	-0.01401700	0.83874410	0.00000000
C	-0.00067560	1.57307490	-1.18388330
C	-0.00067560	1.57307490	1.18388330
C	-0.00349750	2.96074480	-1.18393560
C	-0.00349750	2.96074480	1.18393560
C	-0.00079360	3.69058430	0.00000000
F	0.00175820	3.60713130	-2.35439660
F	0.00175820	3.60713130	2.35439660
F	0.02159090	0.94100780	-2.36304110
F	0.02159090	0.94100780	2.36304110
C	-0.00794910	5.18851650	0.00000000
H	-1.03314600	5.56560100	0.00000000
H	0.49083030	5.57307460	0.88775640
H	0.49083030	5.57307460	-0.88775640

28.

N	-0.01146800	-3.47136640	0.00000000
C	-0.00158070	-4.63922430	0.00000000
C	0.01101700	-6.11923310	0.00000000
F	1.26199970	-6.56773390	0.00000000

F	-0.60861500	-6.58366130	1.08003300
F	-0.60861500	-6.58366130	-1.08003300
Si	-0.02176370	-0.20391440	0.00000000
F	-0.76737760	-0.72810650	1.28062470
F	-0.76737760	-0.72810650	-1.28062470
F	1.46254020	-0.72836360	0.00000000
C	-0.01452860	1.65521020	0.00000000
C	-0.00038120	2.38364630	-1.18856530
C	-0.00038120	2.38364630	1.18856530
C	0.00081000	3.77259410	-1.19166380
C	0.00081000	3.77259410	1.19166380
C	-0.00053980	4.47778660	0.00000000
F	0.00943570	4.42724230	-2.35394820
F	0.00943570	4.42724230	2.35394820
F	0.02175990	1.74647880	-2.36329560
F	0.02175990	1.74647880	2.36329560
H	0.00306050	5.56045160	0.00000000

29.

N	0.00144940	-4.27609250	0.00023930
C	0.01019970	-5.44378150	0.00055210
C	0.02137590	-6.92439860	0.00093760
F	1.27127940	-7.37323640	-0.04050060
F	-0.63435500	-7.38790770	-1.05763600
F	-0.56238390	-7.38680610	1.10130310
Si	-0.01411750	-1.04855970	-0.00111810
F	-0.76166490	-1.56394770	1.28032160
F	-0.75699080	-1.56418080	-1.28517670
F	1.47425260	-1.55706090	0.00163800
C	-0.01052110	0.81496030	-0.00100830
C	-0.00108860	1.54890300	-1.18328680
C	0.00060520	1.54798240	1.18181340
C	-0.00227280	2.93818340	-1.19488870
C	-0.00119810	2.93722840	1.19459150
C	0.02054440	3.65828180	0.00013020
F	-0.03214280	3.55471600	-2.37134340
F	-0.03042870	3.55282000	2.37155450
F	0.00829500	0.92032880	-2.35980830
F	0.01219010	0.91830530	2.35775170
C	0.00554210	5.16986690	0.00080590
F	0.61781130	5.67046870	-1.07246310
F	0.61922010	5.66947440	1.07374170
F	-1.25560100	5.62445270	0.00184950

30.

N	-0.01722380	-3.77543750	0.00000000
C	-0.00421450	-4.94302690	0.00000000
C	0.01214810	-6.42385230	0.00000000
F	1.26430790	-6.86794940	0.00000000
F	-0.60634350	-6.88862200	-1.08007890
F	-0.60634350	-6.88862200	1.08007890
Si	-0.02545680	-0.56126380	0.00000000
F	-0.77341880	-1.07019590	1.28335090
F	-0.77341880	-1.07019590	-1.28335090
F	1.46157020	-1.07237050	0.00000000
C	-0.01547360	1.30420810	0.00000000
C	-0.00004370	2.03228210	-1.18909970
C	-0.00004370	2.03228210	1.18909970
C	0.00184020	3.41974610	-1.20207060
C	0.00184020	3.41974610	1.20207060

C	0.00092920	4.12970080	0.00000000
F	0.01105910	4.07451280	-2.35545740
F	0.01105910	4.07451280	2.35545740
F	0.02177760	1.39753730	-2.36090230
F	0.02177760	1.39753730	2.36090230
C	0.00515950	5.55342420	0.00000000
N	0.00851190	6.72604650	0.00000000

31.

N	0.04563040	-4.73488910	0.00000000
C	0.07777060	-5.90884700	0.00000000
C	0.11558200	-7.28683980	0.00000000
N	0.14763010	-8.46130860	0.00000000
Si	0.01444850	-1.41087810	0.00000000
F	-0.72271460	-1.95540710	1.27953980
F	-0.72271460	-1.95540710	-1.27953980
F	1.49738840	-1.94529090	0.00000000
C	0.00176710	0.43722590	0.00000000
C	0.00539260	1.17694560	-1.18231090
C	0.00539260	1.17694560	1.18231090
C	-0.00250250	2.55980990	-1.18813870
C	-0.00250250	2.55980990	1.18813870
C	-0.01333050	3.29084000	0.00000000
F	-0.01011650	3.23482230	-2.34618730
F	-0.01011650	3.23482230	2.34618730
F	0.02702680	0.54947700	-2.36495330
F	0.02702680	0.54947700	2.36495330
N	0.04724830	4.66420250	0.00000000
H	-0.26415330	5.11224480	-0.84874960
H	-0.26415330	5.11224480	0.84874960

32.

N	0.05300750	-5.21501300	0.00000000
C	0.02600890	-6.38898390	0.00000000
C	-0.00576730	-7.76713580	0.00000000
N	-0.03320330	-8.94174310	0.00000000
Si	0.15941770	-1.91952360	0.00000000
F	-0.60347200	-2.42432150	1.27932080
F	-0.60347200	-2.42432150	-1.27932080
F	1.62079690	-2.50638240	0.00000000
C	0.23007240	-0.06592370	0.00000000
C	0.26931400	0.66483830	-1.18634880
C	0.26931400	0.66483830	1.18634880
C	0.31970810	2.05115130	-1.19765500
C	0.31970810	2.05115130	1.19765500
C	0.35048250	2.76672890	0.00000000
F	0.34413830	2.71112580	-2.35281260
F	0.34413830	2.71112580	2.35281260
F	0.26555500	0.02886010	-2.36340120
F	0.26555500	0.02886010	2.36340120
O	0.46644100	4.11345700	0.00000000
C	-0.78495980	4.82811740	0.00000000
H	-0.82348960	5.44974260	0.89331080
H	-0.82348960	5.44974260	-0.89331080
H	-1.62580400	4.13360890	0.00000000

33.

N	-0.00233700	-4.96082810	0.00056960
C	0.00709920	-6.13508420	-0.00032870
C	0.01819290	-7.51354440	-0.00137080
N	0.02756510	-8.68843240	-0.00225050

Si	-0.01450540	-1.66201020	0.00050900
F	-0.75777980	-2.19511870	1.27988780
F	-0.75751950	-2.19542930	-1.27888780
F	1.46787770	-2.19449740	0.00074920
C	-0.01110610	0.19285680	0.00019150
C	0.00223590	0.92680280	-1.18394910
C	0.00240650	0.92699110	1.18423220
C	-0.00017850	2.31445460	-1.18403840
C	-0.00010780	2.31462250	1.18409750
C	0.00271470	3.04425690	-0.00002580
F	0.00520790	2.96058010	-2.35440530
F	0.00523670	2.96102690	2.35430940
F	0.02407900	0.29413380	-2.36274090
F	0.02456910	0.29446300	2.36310350
C	-0.00405230	4.54216320	0.00003300
H	-1.02918070	4.91942210	0.00226320
H	0.49663780	4.92654190	0.88679300
H	0.49294460	4.92662910	-0.88874160

34.

N	0.00084500	-4.14889880	0.00000000
C	0.01187640	-5.32307140	0.00000000
C	0.02499170	-6.70152000	0.00000000
N	0.03617530	-7.87640060	0.00000000
Si	-0.01895100	-0.86288840	0.00000000
F	-0.76251890	-1.39336840	1.27954970
F	-0.76251890	-1.39336840	-1.27954970
F	1.46523260	-1.38844040	0.00000000
C	-0.01598080	0.99513200	0.00000000
C	-0.00314210	1.72328200	-1.18879190
C	-0.00314210	1.72328200	1.18879190
C	-0.00411880	3.11221440	-1.19181730
C	-0.00411880	3.11221440	1.19181730
C	-0.00654390	3.81721960	0.00000000
F	0.00343820	3.76678790	-2.35390000
F	0.00343820	3.76678790	2.35390000
F	0.01981380	1.08556380	-2.36315130
F	0.01981380	1.08556380	2.36315130
H	-0.00458950	4.89990860	0.00000000

35.

N	-0.00300540	-4.93962830	0.00000000
C	0.00605560	-6.11365150	0.00000000
C	0.01680750	-7.49208610	0.00000000
N	0.02598470	-8.66702330	0.00000000
Si	-0.01718100	-1.68971320	0.00000000
F	-0.76269330	-2.20851050	1.28155980
F	-0.76269330	-2.20851050	-1.28155980
F	1.46854340	-2.20667380	0.00000000
C	-0.00968520	0.17257100	0.00000000
C	0.00202890	0.90571980	-1.18278600
C	0.00202890	0.90571980	1.18278600
C	0.00330070	2.29497200	-1.19488090
C	0.00330070	2.29497200	1.19488090
C	0.02707170	3.01533900	0.00000000
F	-0.02504880	2.91103080	-2.37140600
F	-0.02504880	2.91103080	2.37140600
F	0.01110800	0.27596170	-2.35864310
F	0.01110800	0.27596170	2.35864310
C	0.01507840	4.52716420	0.00000000
F	0.62906210	5.02576120	-1.07310560

F	0.62906210	5.02576120	1.07310560
F	-1.24518510	4.98383200	0.00000000

36.

N	-0.00791770	-4.45420420	0.00000000
C	0.00476650	-5.62813840	0.00000000
C	0.01972780	-7.00648540	0.00000000
N	0.03247420	-8.18141290	0.00000000
Si	-0.02285550	-1.21644830	0.00000000
F	-0.76894860	-1.73168050	1.28222330
F	-0.76894860	-1.73168050	-1.28222330
F	1.46341690	-1.73049180	0.00000000
C	-0.01584910	0.64787620	0.00000000
C	-0.00096580	1.37566140	-1.18931890
C	-0.00096580	1.37566140	1.18931890
C	0.00007370	2.76310420	-1.20221060
C	0.00007370	2.76310420	1.20221060
C	-0.00126490	3.47292380	0.00000000
F	0.00896090	3.41778010	-2.35542730
F	0.00896090	3.41778010	2.35542730
F	0.02114500	0.74035530	-2.36077130
F	0.02114500	0.74035530	2.36077130
C	0.00212880	4.89665980	0.00000000
N	0.00484290	6.06928030	0.00000000

37.

N	0.09778030	-4.12857850	-0.03193440
C	-0.00321790	-5.28844680	-0.15331490
N	-0.04732980	-6.62742220	-0.28881350
H	-0.52296230	-7.09512070	0.47092630
H	-0.37740280	-6.93045840	-1.19522770
Si	0.06838420	-0.99883200	-0.21092190
F	-1.14194210	-1.45050480	0.68972120
F	-0.07097490	-1.61035880	-1.65202200
F	1.42870450	-1.43972780	0.44481470
C	0.04845080	0.85487240	-0.32005620
C	-0.07317480	1.59151830	-1.49640290
C	0.16452680	1.58691890	0.85723610
C	-0.08753010	2.97954620	-1.48181310
C	0.16017520	2.97641140	0.94162560
C	0.03401220	3.64876610	-0.27244810
F	-0.21253390	3.67380620	-2.61222850
F	-0.18891780	0.98346560	-2.68174590
F	0.27690780	0.91708580	2.02559980
N	0.35545120	3.65374920	2.13418820
H	0.09358720	3.12248160	2.95254260
H	-0.01537970	4.59332200	2.14133960
F	0.01338590	4.98750590	-0.26106500

38.

N	-0.09446770	-4.43340050	-0.49219730
C	0.00249560	-5.59983940	-0.50115710
N	0.04145820	-6.94549410	-0.50847900
H	0.41272430	-7.33456630	-1.36469400
H	0.47409590	-7.34176140	0.31498380
Si	0.08927850	-1.32660620	-0.50126110
F	-0.73251790	-1.76767470	0.76353400
F	-0.64650170	-1.78148430	-1.81335950
F	1.54642200	-1.92069080	-0.45031700
C	0.21366180	0.53071890	-0.50971730
C	0.35806680	1.24445330	-1.69830300

C	0.19676170	1.28489400	0.66165800
C	0.44779130	2.62825380	-1.72964950
C	0.28091750	2.67695650	0.69063640
C	0.39916610	3.33411500	-0.53401900
F	0.57921670	3.27796190	-2.88371670
F	0.42502530	0.59588390	-2.86573870
F	0.10841080	0.65974550	1.84454540
O	0.30896500	3.38368040	1.84861700
C	-0.98713860	3.51149170	2.45228230
H	-0.83973150	4.09287460	3.35871340
H	-1.66625810	4.03916230	1.77886170
H	-1.39489040	2.53081130	2.70230230
F	0.47704850	4.66051460	-0.56352510

39.

N	0.15781400	-4.29571820	0.19922390
C	0.04867660	-5.45770060	0.28939050
N	-0.00433730	-6.79924020	0.39106510
H	-0.37103640	-7.12301690	1.27595630
H	-0.44949830	-7.24745570	-0.39834620
Si	0.07822520	-1.16992940	0.35326030
F	-0.02008810	-1.76893590	1.80304580
F	-1.13861320	-1.65422600	-0.52005380
F	1.43716940	-1.58371590	-0.32229770
C	0.01931110	0.68499690	0.44882350
C	0.08712410	1.41942260	-0.73267480
C	-0.08575560	1.43260860	1.61927560
C	0.05558850	2.80499750	-0.82888720
C	-0.12155910	2.81976360	1.60075900
C	-0.04764760	3.48191770	0.38119340
F	-0.22253500	3.51162090	2.73404840
F	0.19161440	0.72970460	-1.88671570
F	-0.15720310	0.83276230	2.80987420
C	0.13835790	3.51033900	-2.14819040
H	1.13016930	3.38771200	-2.58699520
H	-0.58510550	3.09295360	-2.84867990
H	-0.05818370	4.57351580	-2.02924810
F	-0.08248760	4.81762380	0.39617310

40.

N	0.07463130	-3.76274010	0.01170970
C	0.01681050	-4.93206800	0.01157240
N	0.02173430	-6.27869860	0.01149830
H	-0.36776230	-6.68470520	0.85162220
H	-0.36761150	-6.68464600	-0.82873360
Si	0.02741430	-0.60140200	0.01164650
F	-0.72298960	-1.10633760	1.29688790
F	-0.72131040	-1.10497550	-1.27551960
F	1.52113400	-1.09459030	0.01155020
C	0.02699170	1.26102220	0.01096540
C	0.04232160	1.99939900	-1.17127010
C	0.04250530	2.01124150	1.18642890
C	0.04269020	3.38776240	-1.19252680
C	0.04274120	3.39531200	1.22453310
C	0.04098260	4.07088300	0.01645890
F	0.05249880	4.05079890	-2.34547430
F	0.06568910	1.37229510	-2.35045070
F	0.06674780	1.36302290	2.36172810
F	0.04351850	5.40358470	-0.00498630
H	0.05126260	3.93484150	2.16235980

41.

N	0.04201620	-4.25571790	0.33004180
C	-0.00762750	-5.42479120	0.35207750
N	0.00837760	-6.76964570	0.37975860
H	-0.39145080	-7.16392330	1.22051390
H	-0.35439050	-7.19823690	-0.46098600
Si	0.02996390	-1.20945380	0.41630070
F	-0.03389320	-1.74512530	1.89072230
F	-1.23190860	-1.66615550	-0.40238980
F	1.36096050	-1.65418900	-0.29025570
C	0.01876350	0.65774230	0.46237180
C	0.06957180	1.34718490	-0.74880920
C	-0.03634320	1.43140760	1.61527000
C	0.06832020	2.73498060	-0.85444190
C	-0.04074740	2.82008460	1.57170430
C	0.01160100	3.45329860	0.34074230
F	-0.09374180	3.53432700	2.68924780
F	0.12154730	0.59821450	-1.85725180
F	-0.08769480	0.86294230	2.81765090
C	0.12239370	3.51523500	-2.14313390
F	0.17553460	2.73804730	-3.22022210
F	-0.95787930	4.30000980	-2.26360120
F	1.20011770	4.31213640	-2.16573960
F	0.00650920	4.78162760	0.32042930

42.

N	-0.04452060	-3.89188620	-0.19128490
C	0.02112780	-5.06025170	-0.18417180
N	0.02305800	-6.40491710	-0.17594560
H	0.41154900	-6.81754880	-1.01314480
H	0.40418890	-6.80704900	0.66973210
Si	-0.07262800	-0.87850080	-0.16984740
F	-0.82692760	-1.36088890	1.11872250
F	-0.81905560	-1.34684420	-1.46879660
F	1.42961990	-1.34256190	-0.16843710
C	-0.06951590	0.99334710	-0.16415290
C	-0.05281830	1.73375730	-1.34455140
C	-0.05200580	1.73144990	1.01463240
C	-0.05036010	3.12374610	-1.36607610
C	-0.04843790	3.12669430	1.05191330
C	-0.05007110	3.81229350	-0.16342030
F	-0.03990180	3.78379460	-2.51668110
F	-0.02975920	1.11193910	-2.52257300
F	-0.02861430	1.09205350	2.18581790
C	-0.03525690	3.83760760	2.28510560
N	-0.02455670	4.42610200	3.29887290
F	-0.04511360	5.13766350	-0.17571370

43.

N	0.31582510	-3.52488280	-0.00469630
C	0.25964520	-4.69405980	-0.08711100
O	0.23157430	-5.98077220	-0.17577080
C	-1.11490380	-6.51748260	-0.28361870
H	-1.59124430	-6.12582390	-1.18050200
H	-0.98713420	-7.59259390	-0.35167450
H	-1.68440410	-6.24980110	0.60448930
Si	0.28595400	-0.37177270	-0.20696220
F	-0.91769980	-0.82924220	0.70020730
F	0.13409170	-0.98114230	-1.64755670
F	1.64967150	-0.82104280	0.43604900
C	0.26660440	1.48182470	-0.30767980

C	0.13901780	2.22335160	-1.48037360
C	0.38807500	2.20863380	0.87236760
C	0.12372020	3.61130050	-1.45947330
C	0.38282670	3.59773590	0.96291400
C	0.25059700	4.27521960	-0.24769540
F	-0.00795420	4.31062110	-2.58587480
F	0.01794960	1.61999940	-2.66748300
F	0.50710870	1.53338900	2.03690630
N	0.58317970	4.27012510	2.15725290
H	0.32883920	3.73398040	2.97475100
H	0.20986850	5.20867470	2.17159310
F	0.22879180	5.61376030	-0.23005860

44.

N	-0.42934790	-3.84713980	-0.33353920
C	-0.28311710	-5.00850150	-0.41482260
O	-0.15599920	-6.28858260	-0.50667360
C	1.22762400	-6.73160660	-0.44957890
H	1.66094030	-6.44514440	0.50684540
H	1.18381620	-7.81107880	-0.54688880
H	1.78447310	-6.28986610	-1.27381020
Si	-0.19471490	-0.73200230	-0.49445620
F	-1.52552270	-1.07233820	0.27173420
F	-0.23244200	-1.31558650	-1.95246040
F	1.04874460	-1.30034220	0.28596770
C	-0.03503800	1.11860040	-0.57210370
C	0.06005380	1.87116150	-1.74040480
C	-0.00446650	1.82997520	0.62478310
C	0.17348100	3.25490770	-1.71664340
C	0.10608700	3.21595440	0.71026570
C	0.18842340	3.91555330	-0.49450410
F	0.26073240	3.94900810	-2.84902260
F	0.04591750	1.27858770	-2.93686700
F	-0.08000290	1.14366330	1.77728480
O	0.18545890	3.87610220	1.89262870
C	-1.08803400	4.00518300	2.54322570
H	-0.89910620	4.54654650	3.46654540
H	-1.77712790	4.57275460	1.91389380
H	-1.50800260	3.02341950	2.76743060
F	0.28716960	5.24077180	-0.47882950

45.

N	0.36897900	-3.69408330	0.15345020
C	0.30526590	-4.86373570	0.22162980
O	0.26803140	-6.15090100	0.29645180
C	-1.08396090	-6.68053860	0.36714940
H	-1.62544160	-6.41393050	-0.53862430
H	-0.96363380	-7.75594020	0.44390670
H	-1.58431980	-6.28201110	1.24778970
Si	0.27212970	-0.56030830	0.34093850
F	0.15031010	-1.15897300	1.78916580
F	-0.93383690	-1.04772990	-0.54629780
F	1.63959020	-0.98034410	-0.31345360
C	0.21707860	1.29451730	0.42936290
C	0.28623920	2.02326130	-0.75558100
C	0.11962950	2.04753990	1.59699230
C	0.26161340	3.40848270	-0.85780370
C	0.09023240	3.43475810	1.57238020
C	0.16396320	4.09110750	0.34959330
F	-0.00349810	4.13226370	2.70273940
F	0.38605590	1.32757940	-1.90641150

F	0.05038760	1.45282360	2.79024140
C	0.35048120	4.10765840	-2.17999160
H	1.34463560	3.98250320	-2.61293190
H	-0.36941270	3.68755270	-2.88247050
H	0.15438840	5.17152710	-2.06689680
F	0.13509270	5.42692060	0.35867160

46.

N	0.31903380	-3.10384320	0.00920590
C	0.28280940	-4.27664720	0.00981740
O	0.27818210	-5.56635920	0.01071380
C	-1.05949570	-6.13547320	0.00818790
H	-1.58714450	-5.81900330	-0.88957650
H	-0.91021930	-7.20987480	0.01024190
H	-1.59169490	-5.81622410	0.90228220
Si	0.27815100	0.03832510	0.01163270
F	-0.47545850	-0.47207340	1.29289510
F	-0.46138420	-0.46976020	-1.27907060
F	1.77465620	-0.44687350	0.01953810
C	0.26737830	1.89996860	0.01072190
C	0.27983910	2.63846610	-1.17168220
C	0.28335500	2.64998290	1.18630780
C	0.28021170	4.02698590	-1.19319160
C	0.28333910	4.03386050	1.22377390
C	0.28019880	4.71000600	0.01596050
F	0.28958610	4.68922320	-2.34649780
F	0.30159130	2.01122060	-2.35077770
F	0.30937150	2.00210140	2.36189760
F	0.28361380	6.04280160	-0.00382680
H	0.29408000	4.57319020	2.16144640

47.

N	0.27357480	-3.66392800	0.29887390
C	0.23955380	-4.83649830	0.31260980
O	0.23858750	-6.12527550	0.32806510
C	-1.09752270	-6.69948670	0.34923210
H	-0.94373140	-7.77294990	0.37375250
H	-1.62318800	-6.36223180	1.24055430
H	-1.63316370	-6.40441340	-0.55104100
Si	0.24920200	-0.59256660	0.39566040
F	0.18454470	-1.12753280	1.87016510
F	-1.01033820	-1.06103830	-0.42054740
F	1.58069070	-1.04072000	-0.30736640
C	0.23335750	1.27404510	0.43539530
C	0.28346060	1.96011780	-0.77778120
C	0.17744450	2.05070980	1.58636400
C	0.28075260	3.34759410	-0.88714140
C	0.17246780	3.43923340	1.53896230
C	0.22439710	4.06905980	0.30616930
F	0.11905480	4.15651160	2.65442730
F	0.33615580	1.20798120	-1.88396860
F	0.12612200	1.48526430	2.79006930
C	0.33102950	4.12449570	-2.17814390
F	0.38499110	3.34439140	-3.25301380
F	-0.75206040	4.90532850	-2.29907130
F	1.40606760	4.92470640	-2.20443390
F	0.21854990	5.39720240	0.28220840

48.

N	-0.30826070	-3.29594780	-0.16801390
C	-0.24958820	-4.46757410	-0.16438150

O	-0.22307220	-5.75596460	-0.16103300
C	1.12387080	-6.30564920	-0.15908200
H	1.64667860	-5.98052300	0.73825830
H	0.99048740	-7.38216640	-0.16073330
H	1.65025210	-5.97828920	-1.05353650
Si	-0.32129000	-0.23983100	-0.15637740
F	-1.07911420	-0.71938080	1.13077120
F	-1.07362810	-0.70557380	-1.45263160
F	1.17302860	-0.72956820	-0.15717860
C	-0.29987550	1.63084620	-0.14946610
C	-0.28354290	2.37147020	-1.32976960
C	-0.28218500	2.36788730	1.03008070
C	-0.28353360	3.76142910	-1.35067510
C	-0.28161950	3.76311720	1.06740270
C	-0.28570750	4.44924830	-0.14765490
F	-0.27460250	4.42247360	-2.50051580
F	-0.25970400	1.74943040	-2.50755040
F	-0.25705770	1.72695080	2.20024590
C	-0.27165520	4.47569680	2.29943840
N	-0.26449200	5.06742170	3.31134070
F	-0.28538880	5.77449650	-0.15893840

49.

N	-0.0671287	-3.8418800	-0.1981154
C	-0.0340181	-5.0088982	-0.2199380
C	0.0066650	-6.4644790	-0.2455627
H	-0.4945305	-6.8654827	0.6344276
H	1.0402923	-6.8080447	-0.2516566
H	-0.4948722	-6.8353764	-1.1384822
Si	-0.0270332	-0.6660874	-0.2417293
F	-0.8100737	-1.1421964	1.0368132
F	-0.7594590	-1.1802125	-1.5341934
F	1.4519729	-1.2053494	-0.2033151
C	0.0083592	1.1913019	-0.2662961
C	-0.0027739	1.9212451	-1.4542779
C	0.0631747	1.9328156	0.9101858
C	0.0021949	3.3083502	-1.4583032
C	0.0714743	3.3247189	0.9754289
C	0.0410994	3.9879899	-0.2491788
F	-0.0214277	3.9909962	-2.6020019
F	-0.0193094	1.2949581	-2.6369761
F	0.1033784	1.2890588	2.0952410
N	0.1949893	4.0103414	2.1730893
H	-0.1250949	3.4868452	2.9755069
H	-0.1677793	4.9527219	2.1497279
F	0.0399003	5.3266637	-0.2503939

50.

N	-0.0215872	-4.1687629	-0.4968006
C	0.0081722	-5.3360385	-0.4940826
C	0.0453239	-6.7919039	-0.4898812
H	1.0781375	-7.1378020	-0.4870862
H	-0.4553748	-7.1793396	-1.3761766
H	-0.4583820	-7.1739434	0.3970849
Si	0.1361575	-1.0091866	-0.4881893
F	-0.6825313	-1.4670458	0.7732045
F	-0.6007683	-1.4678097	-1.7985945
F	1.5932608	-1.6026273	-0.4417055
C	0.2504671	0.8478502	-0.4890274
C	0.3809337	1.5681147	-1.6754388
C	0.2384916	1.5958504	0.6865649

C	0.4625634	2.9525978	-1.7005985
C	0.3182292	2.9878273	0.7215743
C	0.4224097	3.6517492	-0.5007073
F	0.5792906	3.6087204	-2.8524382
F	0.4405537	0.9257154	-2.8464646
F	0.1581881	0.9643704	1.8661922
O	0.3526997	3.6858661	1.8847402
C	-0.9490880	3.8530798	2.4660289
H	-0.7988270	4.4156254	3.3839012
H	-1.5955708	4.4145397	1.7876484
H	-1.3965086	2.8839255	2.6936792
F	0.4937593	4.9786274	-0.5234273

51.

N	0.0046583	-4.0116619	0.3609747
C	0.0196931	-5.1792037	0.3621025
C	0.0387393	-6.6353638	0.3630901
H	-0.4681959	-7.0134517	1.2498987
H	-0.4681933	-7.0144403	-0.5233061
H	1.0672751	-6.9938120	0.3633024
Si	-0.0211148	-0.8546796	0.3669240
F	-0.7622191	-1.3667819	1.6554665
F	-0.7734609	-1.3623483	-0.9167036
F	1.4722079	-1.3540121	0.3594515
C	-0.0232606	1.0044197	0.3734727
C	-0.0091355	1.7542822	-0.8014713
C	-0.0107123	1.7385538	1.5578651
C	-0.0208178	3.1433643	-0.8673629
C	-0.0157504	3.1249899	1.5679440
C	-0.0200769	3.8034190	0.3556668
F	-0.0059856	3.7986089	2.7160192
F	0.0236987	1.0952421	-1.9744921
F	0.0146592	1.1124073	2.7383899
C	-0.0102301	3.8793322	-2.1724214
H	0.9976984	3.9074703	-2.5912735
H	-0.6557584	3.3811137	-2.8943869
H	-0.3516668	4.9035171	-2.0377539
F	-0.0220518	5.1390351	0.3886037

52.

N	0.0004055	-3.3992711	0.0111120
C	0.0099836	-4.5667424	0.0110123
C	0.0219221	-6.0228040	0.0109576
H	-0.4869411	-6.3985820	0.8976340
H	-0.4869349	-6.3985462	-0.8757340
H	1.0487623	-6.3860346	0.0109438
Si	-0.0229282	-0.2783295	0.0110172
F	-0.7720421	-0.7807050	1.2977895
F	-0.7707187	-0.7797908	-1.2773504
F	1.4712767	-0.7732938	0.0109028
C	-0.0209225	1.5827900	0.0103776
C	-0.0057882	2.3208519	-1.1720263
C	-0.0056681	2.3325879	1.1860139
C	-0.0056499	3.7092224	-1.1932675
C	-0.0056109	3.7164587	1.2237909
C	-0.0073694	4.3923693	0.0158482
F	0.0039954	4.3713526	-2.3465452
F	0.0177861	1.6934667	-2.3510494
F	0.0186451	1.6845181	2.3614580
F	-0.0047748	5.7251219	-0.0046466
H	0.0025720	4.2553597	2.1617614

53.

N	-0.0062919	-3.9578641	0.3128875
C	0.0056482	-5.1249582	0.3336199
C	0.0203514	-6.5807194	0.3606189
H	0.5402812	-6.9635360	-0.5165495
H	0.5316781	-6.9302211	1.2565839
H	-0.9997327	-6.9624980	0.3628999
Si	-0.0227874	-0.8893891	0.4052324
F	-0.0857537	-1.4268337	1.8790817
F	-1.2837384	-1.3502476	-0.4120428
F	1.3070686	-1.3432721	-0.2989888
C	-0.0290547	0.9767665	0.4479381
C	0.0234639	1.6638079	-0.7645316
C	-0.0813772	1.7521386	1.5998907
C	0.0261290	3.0513317	-0.8723561
C	-0.0817301	3.1406910	1.5539707
C	-0.0280738	3.7716342	0.3217762
F	-0.1323670	3.8569297	2.6701768
F	0.0735937	0.9124774	-1.8714029
F	-0.1335471	1.1852921	2.8028759
C	0.0828784	3.8292495	-2.1624851
F	0.1319828	3.0498342	-3.2381284
F	-0.9938301	4.6185610	-2.2835083
F	1.1643066	4.6210536	-2.1868527
F	-0.0290979	5.0997720	0.2992938

54.

N	-0.0092221	-3.5883362	-0.1724564
C	0.0058474	-4.7554976	-0.1705352
C	0.0249543	-6.2114410	-0.1689503
H	-0.4820609	-6.5887422	0.7181337
H	-0.4820578	-6.5902444	-1.0554010
H	1.0536805	-6.5693082	-0.1686260
Si	-0.0282777	-0.5429303	-0.1627307
F	-0.7810897	-1.0273047	1.1262315
F	-0.7801023	-1.0172444	-1.4567744
F	1.4704121	-1.0171739	-0.1654900
C	-0.0223810	1.3274938	-0.1582977
C	-0.0059590	2.0683940	-1.3383801
C	-0.0060135	2.0643494	1.0212804
C	-0.0046982	3.4583800	-1.3581531
C	-0.0043862	3.4594272	1.0599859
C	-0.0062860	4.1458794	-0.1547781
F	0.0054512	4.1201464	-2.5076100
F	0.0174881	1.4470104	-2.5165531
F	0.0177487	1.4234234	2.1914549
C	0.0061254	4.1684255	2.2943026
N	0.0142369	4.7542193	3.3096604
F	-0.0034102	5.4710743	-0.1663132

55.

N	-0.06062510	-4.95272560	-0.24308310
C	-0.02037380	-6.11693030	-0.27447440
H	0.01669800	-7.18318510	-0.30371700
Si	-0.02825570	-1.69492940	-0.27758470
F	-0.80465390	-2.18572280	0.99977540
F	-0.76219600	-2.22043890	-1.56502320
F	1.44668180	-2.24633960	-0.24371870
C	0.00587790	0.15961650	-0.30043960
C	-0.00270070	0.88841500	-1.48929430

C	0.06049880	0.90051380	0.87661790
C	0.00596380	2.27537360	-1.49368400
C	0.07240450	2.29240630	0.94098840
C	0.04588420	2.95496750	-0.28442540
F	-0.01457970	2.95748790	-2.63731600
F	-0.02027800	0.26048320	-2.67094420
F	0.09736430	0.25593680	2.06111030
N	0.19687000	2.97873260	2.13744870
H	-0.12523760	2.45895940	2.94145840
H	-0.15809780	3.92403160	2.11221440
F	0.04875500	4.29334720	-0.28590900

56.

N	-0.01314710	-5.21938330	-0.57681840
C	0.05693420	-6.38250380	-0.57345380
H	0.12151570	-7.44790630	-0.57082980
Si	0.12879970	-1.99098090	-0.56151650
F	-0.69704300	-2.46270310	0.69040500
F	-0.59796790	-2.45939120	-1.87446150
F	1.58041410	-2.59775600	-0.50534000
C	0.24508260	-0.13698870	-0.55793730
C	0.37926340	0.58617320	-1.74225150
C	0.23496980	0.60628470	0.62076590
C	0.46714760	1.97025180	-1.76229720
C	0.32182020	1.99755710	0.66050600
C	0.42968000	2.66507350	-0.55968680
F	0.58796770	2.62970970	-2.91136750
F	0.43661690	-0.05325670	-2.91477410
F	0.15065090	-0.03014810	1.79724990
O	0.36075740	2.69121440	1.82562210
C	-0.93984000	2.87003300	2.40667320
H	-0.78390480	3.43058420	3.32474660
H	-1.58122810	3.43807050	1.72902840
H	-1.39598480	1.90474310	2.63324710
F	0.50749580	3.99132310	-0.57750980

57.

N	0.05527310	-5.08190810	0.27228670
C	0.03527090	-6.24139030	0.38657290
H	0.01665220	-7.30332390	0.49203840
Si	0.00574810	-1.84613570	0.39524820
F	-0.08269770	-2.45315220	1.84251830
F	-1.21894170	-2.33810340	-0.46255960
F	1.34864770	-2.29855920	-0.28980520
C	-0.02901590	0.00606460	0.48366330
C	0.04161720	0.73629340	-0.70041260
C	-0.12035090	0.75615500	1.65391010
C	0.02455270	2.12178530	-0.79891570
C	-0.14049200	2.14336870	1.63250290
C	-0.06519510	2.80176130	0.41074870
F	-0.22839520	2.83847650	2.76423390
F	0.13284370	0.04127590	-1.85215240
F	-0.19361980	0.15781350	2.84473060
C	0.10778520	2.82505160	-2.11929950
H	1.10267490	2.71027510	-2.55330320
H	-0.60901050	2.40016810	-2.82195130
H	-0.09770490	3.88685190	-2.00315900
F	-0.08564170	4.13723190	0.42310450

58.

N	-0.00086580	-4.56658800	0.01217560

C	0.01262790	-5.73171000	0.01140600
H	0.02515450	-6.79898490	0.01078370
Si	-0.01713340	-1.34380400	0.01394500
F	-0.77334010	-1.86042620	1.29119490
F	-0.75282120	-1.85880600	-1.27631550
F	1.47125870	-1.85647030	0.02435900
C	-0.01431400	0.51429120	0.01315800
C	-0.00259510	1.25084140	-1.17037390
C	0.00227300	1.26383770	1.18906130
C	-0.00323080	2.63901960	-1.19207580
C	0.00051320	2.64746310	1.22574200
C	-0.00428370	3.32233290	0.01713330
F	0.00427770	3.30085200	-2.34505080
F	0.01805910	0.62131530	-2.34797750
F	0.02931330	0.61494530	2.36371920
F	-0.00396490	4.65458370	-0.00404510
H	0.00907160	3.18730730	2.16316040

59.

N	-0.00166470	-5.04922480	0.36705650
C	0.00582790	-6.21388500	0.39425420
H	0.01271490	-7.28100920	0.41985120
Si	-0.01774510	-1.88256210	0.45347780
F	-0.08180610	-2.43588910	1.92161460
F	-1.27310240	-2.36004770	-0.36318240
F	1.30754630	-2.35362690	-0.24832920
C	-0.02421110	-0.01973370	0.49763600
C	0.02786010	0.66837130	-0.71430410
C	-0.07685330	0.75302620	1.65151230
C	0.02927330	2.05584870	-0.81970270
C	-0.07843420	2.14146820	1.60747160
C	-0.02524050	2.77400360	0.37577030
F	-0.12953970	2.85597180	2.72425640
F	0.07884640	-0.08247370	-1.82131300
F	-0.12816610	0.18273050	2.85260630
C	0.08671030	2.83585690	-2.10894480
F	0.13090810	2.05737060	-3.18533100
F	-0.98718230	3.62894350	-2.22650460
F	1.17115000	3.62312790	-2.13329790
F	-0.02689160	4.10173300	0.35540240

60.

N	-0.00535090	-4.72087830	-0.20666650
C	0.01021340	-5.88561850	-0.19725120
H	0.02468250	-6.95295900	-0.18854340
Si	-0.02233720	-1.58291700	-0.19234560
F	-0.77584930	-2.08686910	1.08883430
F	-0.76704190	-2.06983730	-1.48599220
F	1.47046380	-2.07564420	-0.19259230
C	-0.01674510	0.28381930	-0.18338210
C	-0.00196090	1.02409640	-1.36396450
C	-0.00014550	1.01911600	0.99731610
C	-0.00054690	2.41378020	-1.38360920
C	0.00123300	2.41403390	1.03574480
C	-0.00152840	3.10027650	-0.17944270
F	0.00888580	3.07545510	-2.53261080
F	0.01967270	0.40136920	-2.54116040
F	0.02376990	0.37609060	2.16613430
C	0.01179460	3.12527600	2.26889490
N	0.01969640	3.71635660	3.28118450
F	0.00109400	4.42505360	-0.19054820

61.

N	-0.03449920	-3.90714460	-0.10266280
C	-0.01656260	-5.07335850	-0.16597840
C	0.00572840	-6.55072850	-0.24490830
F	-0.60942270	-7.07634700	0.80977360
F	-0.61239910	-6.96203110	-1.34722650
F	1.25951890	-6.99109800	-0.26974590
Si	-0.02415240	-0.60260400	-0.23363700
F	-1.24413330	-1.07813810	0.64250150
F	-0.14992590	-1.22112450	-1.67365730
F	1.31079460	-1.10710780	0.43275510
C	-0.01245310	1.24582640	-0.31981990
C	-0.11426070	1.99315380	-1.49174180
C	0.10586130	1.96360480	0.86637350
C	-0.10704640	3.38079670	-1.46396830
C	0.12206370	3.35208850	0.96300080
C	0.01568320	4.03643590	-0.24698460
F	-0.21323890	4.08705020	-2.58766160
F	-0.23039080	1.39456490	-2.68113130
F	0.19930910	1.27861550	2.02724870
N	0.31830060	4.01623070	2.16112300
H	0.04787750	3.48387820	2.97578710
H	-0.03266590	4.96326100	2.17425920
F	0.01601390	5.37417560	-0.22369880

62.

N	-0.00532740	-4.24132030	-0.51417910
C	0.01379920	-5.40908450	-0.50097260
C	0.03766800	-6.88868470	-0.48353090
F	-0.57797270	-7.34490590	0.60249380
F	-0.57919090	-7.37106070	-1.55738020
F	1.29182990	-7.32803700	-0.47897010
Si	0.14822570	-0.95791980	-0.50476200
F	-0.67534410	-1.44777160	0.74254960
F	-0.57283050	-1.44153780	-1.81612410
F	1.59736480	-1.57184150	-0.44826460
C	0.25328810	0.89377090	-0.49748830
C	0.37563300	1.62161190	-1.68033400
C	0.24183800	1.63185560	0.68462720
C	0.45218560	3.00637470	-1.69527200
C	0.31786890	3.02346650	0.72915140
C	0.41493250	3.69601910	-0.48942470
F	0.56132140	3.67126890	-2.84186250
F	0.43130760	0.98572230	-2.85460340
F	0.16620980	0.98866200	1.85767520
O	0.35571220	3.71155840	1.89710530
C	-0.94712870	3.89585360	2.47228720
H	-0.79195520	4.44389020	3.39795450
H	-1.57864550	4.47763160	1.79707310
H	-1.41327400	2.93199930	2.68470780
F	0.48248440	5.02247890	-0.50245640

63.

N	-0.5354870	-4.1091763	0.0000000
C	-0.4864462	-5.2762050	0.0000000
C	-0.4214770	-6.7545364	0.0000000
Si	-0.3714627	-0.8069797	0.0000000
F	-1.1458163	-1.2908627	-1.2830658
F	1.0706118	-1.4331623	0.0000000
F	-1.1458163	-1.2908627	1.2830658

C	-0.2855790	1.0438506	0.0000000
C	0.8825945	1.8064389	0.0000000
C	-1.4825356	1.7554319	0.0000000
C	0.9139805	3.1975130	0.0000000
C	-1.5257785	3.1402618	0.0000000
C	-0.3226998	3.8336654	0.0000000
F	-2.6850184	3.7919855	0.0000000
F	2.0618629	1.1625979	0.0000000
F	-2.6424060	1.0872704	0.0000000
C	2.1820248	3.9965985	0.0000000
H	2.2295467	4.6399084	0.8798142
H	3.0479324	3.3394419	0.0000000
H	2.2295467	4.6399084	-0.8798142
F	-0.3643710	5.1682257	0.0000000
F	-1.6469320	-7.2688906	0.0000000
F	0.2218628	-7.1862113	-1.0800013
F	0.2218628	-7.1862113	1.0800013

64.

N	-0.00839010	-3.48731300	0.00824350
C	0.00529480	-4.65520110	0.00938870
C	0.02192630	-6.13501270	0.01038750
F	-0.59650720	-6.60180010	-1.06936210
F	1.27407520	-6.58042290	0.01073640
F	-0.59651840	-6.60050060	1.09077210
Si	-0.02016050	-0.20710160	0.01262010
F	-0.77911310	-0.73674020	1.28369670
F	-0.74862020	-0.73359680	-1.27770230
F	1.46360590	-0.73488720	0.02840020
C	-0.01735690	1.64792200	0.01206650
C	-0.00778220	2.38339710	-1.17236350
C	-0.00035920	2.39682420	1.18848820
C	-0.00862770	3.77129050	-1.19461230
C	-0.00310700	3.78025380	1.22428250
C	-0.00911240	4.45436860	0.01494750
F	-0.00230090	4.43254500	-2.34733630
F	0.01036020	1.75256700	-2.34892730
F	0.02742980	1.74655730	2.36217920
F	-0.01000450	5.78596170	-0.00721290
H	0.00526840	4.32088890	2.16130740

65.

N	-0.04041180	-4.05404300	0.32955840
F	-1.21616730	-7.19028340	0.38026440
C	-0.01611060	-5.22138200	0.34832610
C	0.01924110	-6.70123310	0.37191900
F	0.65332450	-7.16117080	-0.70132830
F	0.65429110	-7.12694580	1.45874290
Si	-0.01692290	-0.82618680	0.39865390
F	-0.09450720	-1.38922250	1.86308130
F	-1.26177010	-1.31612560	-0.42748030
F	1.30875750	-1.31921010	-0.28894990
C	-0.02052530	1.03339020	0.44109280
C	0.03071070	1.72085200	-0.77128600
C	-0.07435710	1.80518000	1.59578510
C	0.02945590	3.10815660	-0.87623480
C	-0.07692290	3.19340410	1.55166580
C	-0.02607760	3.82589970	0.31957890
F	-0.12931580	3.90762580	2.66797590
F	0.08330940	0.96850780	-1.87718380
F	-0.12629860	1.23339500	2.79582680

C	0.07721680	3.88889210	-2.16584290
F	0.13788700	3.11040040	-3.24137350
F	-1.01071190	4.66220380	-2.28540250
F	1.14744140	4.69475610	-2.18719480
F	-0.03153630	5.15313940	0.29980520

66.

N	-0.01436610	-3.68507020	-0.17766070
C	0.00259450	-4.85264200	-0.17424980
C	0.02531630	-6.33324500	-0.16919850
F	-0.59115730	-6.79706350	0.91238180
F	1.27944790	-6.77183490	-0.16771510
F	-0.59100800	-6.80468580	-1.24773200
Si	-0.02695410	-0.47081480	-0.16775260
F	-0.78109430	-0.99063520	1.10728190
F	-0.76544180	-0.97200770	-1.46009480
F	1.46055320	-0.98073440	-0.16515870
C	-0.02138600	1.39230670	-0.15743760
C	-0.00787990	2.13291870	-1.33795270
C	-0.00443300	2.12525870	1.02482850
C	-0.00632260	3.52240820	-1.35573510
C	-0.00332530	3.51986090	1.06461880
C	-0.00666910	4.20704660	-0.15018270
F	0.00240960	4.18537250	-2.50339680
F	0.01212820	1.51006660	-2.51484330
F	0.01982140	1.47925950	2.19177760
C	0.00716730	4.23063540	2.29811470
N	0.01473780	4.82228260	3.31008620
F	-0.00413860	5.53131710	-0.15997930

67.

N	-0.04164150	-4.54787770	-0.11980670
C	-0.02293670	-5.71988320	-0.19160240
C	-0.00088600	-7.09569650	-0.27441260
N	0.01777020	-8.26842950	-0.34389360
Si	-0.01626900	-1.24357770	-0.25502320
F	-1.22809760	-1.71954160	0.63261510
F	-0.15630510	-1.86656280	-1.69222870
F	1.32305510	-1.75398440	0.39882360
C	-0.00394670	0.60357250	-0.34285890
C	-0.10538760	1.34963390	-1.51563300
C	0.10994390	1.32186200	0.84348830
C	-0.10401420	2.73725180	-1.48818230
C	0.12003990	2.71036260	0.93968330
C	0.01315870	3.39365790	-0.27099400
F	-0.21017870	3.44264710	-2.61219450
F	-0.21616410	0.74966350	-2.70472990
F	0.20456010	0.63695640	2.00432390
N	0.31096070	3.37571920	2.13767350
H	0.04339650	2.84195140	2.95235270
H	-0.04449810	4.32108980	2.15063400
F	0.00744030	4.73118500	-0.24803450

68.

N	-0.00149650	-4.87082150	-0.55384650
C	0.01988710	-6.04486170	-0.54402620
C	0.04479100	-7.42309640	-0.53135980
N	0.06555610	-8.59781230	-0.51994840
Si	0.15025100	-1.57187890	-0.54148650
F	-0.67801540	-2.06404890	0.70217880
F	-0.56497000	-2.06112660	-1.85422500

F	1.59735000	-2.19062070	-0.47800870
C	0.25597190	0.27858390	-0.53655580
C	0.37976930	1.00521690	-1.72006890
C	0.24383230	1.01713730	0.64530850
C	0.45685660	2.38990240	-1.73596760
C	0.32100600	2.40860920	0.68869700
C	0.41895070	3.08016350	-0.53047020
F	0.56718800	3.05401320	-2.88269120
F	0.43619990	0.36798930	-2.89348530
F	0.16621220	0.37413540	1.81825240
O	0.35881770	3.09726540	1.85609930
C	-0.94545680	3.29284100	2.42461460
H	-0.79069300	3.84217220	3.34956450
H	-1.56951940	3.87717680	1.74483940
H	-1.41920890	2.33264010	2.63689150
F	0.48672040	4.40642020	-0.54430590

69.

N	0.5139411	-4.7562066	0.0000000
C	0.4429566	-5.9284002	0.0000000
C	0.3578157	-7.3043939	0.0000000
N	0.2837210	-8.4769710	0.0000000
Si	0.3954165	-1.4401514	0.0000000
F	-1.0533253	-2.0516666	0.0000000
F	1.1630847	-1.9385453	-1.2818614
F	1.1630847	-1.9385453	1.2818614
C	0.3319472	0.4108391	0.0000000
C	1.5373437	1.1083991	0.0000000
C	-0.8277385	1.1865855	0.0000000
C	1.5962682	2.4927331	0.0000000
C	-0.8430763	2.5778383	0.0000000
C	0.4009201	3.1995045	0.0000000
F	2.7620236	3.1324046	0.0000000
F	2.6889134	0.4259658	0.0000000
F	-2.0142088	0.5563415	0.0000000
F	0.4577799	4.5332729	0.0000000
C	-2.1012488	3.3925138	0.0000000
H	-2.1400913	4.0360598	0.8801782
H	-2.9754361	2.7463625	0.0000000
H	-2.1400913	4.0360598	-0.8801782

70.

N	0.3112442	-4.1591477	0.0000000
C	0.3245513	-5.3333876	0.0000000
Si	0.0232061	-0.8640578	0.0000000
F	-1.3959950	-1.5396743	0.0000000
F	0.8141140	-1.3200224	-1.2827376
F	0.8141140	-1.3200224	1.2827376
C	-0.1313840	0.9827099	0.0000000
C	1.0358228	1.7447153	0.0000000
C	-1.3252446	1.7023258	0.0000000
C	1.0350785	3.1313760	0.0000000
C	-1.3843549	3.0862730	0.0000000
C	-0.1905433	3.7870554	0.0000000
F	2.1735783	3.8161365	0.0000000
F	2.2189463	1.1204941	0.0000000
F	-2.4881182	1.0362543	0.0000000
F	-0.1994176	5.1187574	0.0000000
H	-2.3316674	3.6087834	0.0000000
C	0.3407577	-6.7118604	0.0000000
N	0.3553119	-7.8867086	0.0000000

71.

Si	0.2262462	-1.5121037	0.0000000
F	0.9359415	-2.0780604	-1.2847629
F	0.9359415	-2.0780604	1.2847629
F	-1.2829165	-1.9449709	0.0000000
C	0.3824208	0.3410701	0.0000000
C	1.6618767	0.8852333	0.0000000
C	-0.6828427	1.2412231	0.0000000
C	1.8939753	2.2515118	0.0000000
C	-0.5097432	2.6248748	0.0000000
C	0.8005896	3.1042849	0.0000000
N	-0.0454879	-4.7777191	0.0000000
C	-0.2981993	-5.9243749	0.0000000
F	3.1269705	2.7383031	0.0000000
F	2.7187337	0.0704831	0.0000000
F	-1.9179681	0.7382985	0.0000000
C	-0.5949983	-7.2706276	0.0000000
N	-0.8477129	-8.4181047	0.0000000
C	-1.6243940	3.6428726	0.0000000
F	1.0314730	4.4111782	0.0000000
F	-2.8338762	3.0929031	0.0000000
F	-1.5380149	4.4308926	1.0804350
F	-1.5380149	4.4308926	-1.0804350

72.

Si	0.1163620	-1.1260500	0.0000000
F	-1.3654834	-1.6430493	0.0000000
F	0.8622498	-1.6394262	-1.2853346
F	0.8622498	-1.6394262	1.2853346
C	0.1444863	0.7344442	0.0000000
C	1.3806196	1.3770051	0.0000000
C	-0.9758379	1.5576792	0.0000000
C	1.5183559	2.7582633	0.0000000
C	-0.8954777	2.9518610	0.0000000
C	0.3709963	3.5381343	0.0000000
N	0.0771947	-4.3709919	0.0000000
C	-0.0375519	-5.5394198	0.0000000
F	2.7164446	3.3241689	0.0000000
F	2.4939407	0.6419554	0.0000000
F	-2.1937270	1.0178274	0.0000000
C	-0.1740512	-6.9111651	0.0000000
N	-0.2916555	-8.0802571	0.0000000
F	0.4872663	4.8572171	0.0000000
C	-2.0667919	3.7612464	0.0000000
N	-3.0295894	4.4299833	0.0000000

Cartesian coordinates of the selected CSD structures

AHESIR

Si	1.69200000	4.48700000	0.57500000
F	2.37200000	3.07100000	0.80300000
F	1.88200000	5.11300000	2.00700000
F	2.69100000	5.14000000	-0.44300000
C	-0.92600000	5.01100000	-0.35900000
C	0.00100000	4.01800000	-0.01100000
C	-0.39500000	2.70300000	-0.14500000
H	0.21600000	2.01200000	0.08600000
C	-1.67300000	2.35100000	-0.61200000

C	-2.55900000	3.36800000	-0.94900000
H	-3.42500000	3.14700000	-1.27000000
C	-2.19800000	4.69400000	-0.82500000
H	-2.81200000	5.38300000	-1.05500000
Si	1.51800000	1.97600000	4.36800000
F	2.19800000	3.39200000	4.59700000
F	1.70800000	1.34900000	5.80000000
F	2.51700000	1.32200000	3.35000000
C	-1.10000000	1.45100000	3.43500000
C	-0.17300000	2.44400000	3.78200000
C	-0.56900000	3.76000000	3.64900000
H	0.04200000	4.45000000	3.88000000
C	-1.84700000	4.11100000	3.18100000
C	-2.73300000	3.09400000	2.84400000
H	-3.59900000	3.31500000	2.52300000
C	-2.37200000	1.76800000	2.96800000
H	-2.98600000	1.07900000	2.73800000
H	-0.83718663	0.41868120	3.53573250
H	-0.66338353	6.04330219	-0.25758615
H	-2.14418666	5.13480094	3.08928450
H	-1.97018666	1.32719906	-0.70371550

WIZHUI01

C	0.73700000	12.25900000	13.66100000
C	0.03300000	13.29800000	14.23600000
H	-0.73100000	13.62000000	13.81600000
C	1.90900000	11.77700000	14.30000000
C	1.55500000	13.38500000	16.07800000
H	1.80800000	13.75800000	16.89100000
C	0.44100000	13.87000000	15.42500000
H	-0.03500000	14.58300000	15.78600000
Si	2.93500000	10.37900000	13.59800000
F	4.43800000	10.73900000	13.57500000
F	2.55200000	10.01100000	12.14900000
C	2.30500000	12.34600000	15.53700000
F	2.74400000	9.10400000	14.45600000
Si	5.14500000	6.58900000	11.78300000
F	4.69100000	7.05500000	10.37700000
F	5.05500000	7.84600000	12.70000000
C	4.61500000	4.62300000	13.72800000
C	3.03600000	4.63900000	11.86800000
C	3.89000000	3.59600000	14.30500000
H	4.15600000	3.25400000	15.12900000
C	4.19200000	5.17600000	12.49200000
C	2.36700000	3.58900000	12.47500000
H	1.62000000	3.22600000	12.05800000
C	2.78000000	3.07100000	13.67800000
H	2.31300000	2.36800000	14.06700000
F	6.65400000	6.26200000	11.62000000
H	3.16802479	11.98581197	16.05695461
H	0.36598385	11.83579854	12.75097388
H	2.66423696	5.03078105	10.94431182
H	5.49808148	4.98119771	14.21458144
